# Dynamics in Quality of Life of Breast Cancer Patients Following Surgery: Systematic Review and Meta-Analysis

**DOI:** 10.3390/cancers17193108

**Published:** 2025-09-24

**Authors:** Iryna Makhnevych, Mussab Ibrahim Mohamed Fadl Elseed, Ibrahim Mohamed Ahmed Musa, Jood Jasem Shaddad Alblooshi, Darya Smetanina, Faisal Tahsin, Yauhen Statsenko

**Affiliations:** 1 Psychiatry Department, Cleveland Clinic Abu Dhabi, Abu Dhabi P.O. Box 112412, United Arab Emirates; 2 Department of Radiology, College of Medicine and Health Sciences, United Arab Emirates University, Abu Dhabi P.O. Box 15551, United Arab Emirates; musab_i_m@live.com (M.I.M.F.E.); hema2030hema@gmail.com (I.M.A.M.); 201904089@uaeu.ac.ae (J.J.S.A.); daryasm@uaeu.ac.ae (D.S.); f.tahsin11@gmail.com (F.T.)

**Keywords:** breast cancer, global quality of life, surgery type, mastectomy, breast-conserving surgery, reconstruction, meta-analysis, trajectory modeling, survivorship, age differences

## Abstract

**Background and Objectives:** Surgical treatment is central to breast cancer management; however, its long-term impact on QoL varies substantially among patients. This study sought to model the dynamic trajectories of postoperative QoL following breast-conserving surgery (BCS), mastectomy with immediate reconstruction (Mx+IR), and mastectomy alone (MA). It also examined how these trajectories varied across different age groups and over time. **Materials and Methods:** The review and meta-analysis identified 150 peer-reviewed studies reporting QoL outcomes using validated instruments (EORTC QLQ-C30 or BREAST-Q). A total of 123 observations from 45 studies were included for analysis of global QoL. We standardized QoL scores to a 0–100 scale and harmonized postoperative assessments across six time intervals, extending to more than 73 months. Multilevel random-effects models evaluated linear, quadratic, and logarithmic functions. Subgroup analyses and meta-regressions assessed the moderating effects of surgical type and age. **Results:** BCS showed the steepest QoL gains, followed by Mx+IR, while MA had the lowest scores and slowest recovery. Compared to BCS, MA showed significantly poorer and delayed recovery, and Mx+IR showed a smaller, borderline decrease. All groups displayed modest long-term QoL plateauing. **Conclusions:** Global QoL after breast cancer surgery follows distinct, time-dependent patterns shaped by surgical approach and age. These findings emphasize the importance of discussing patients’ quality-of-life expectations with them so that survivorship care can be personalized to their needs.

## Simple Summary

Surgical treatment is a cornerstone in breast cancer management, yet its long-term influence on quality of life (QoL) remains variable and not fully understood. This study examined the dynamic patterns of global QoL following different surgical approaches, including breast-conserving surgery, mastectomy, and breast reconstruction. By tracking overall QoL changes over time, this research aimed to generate clinically meaningful insights into how surgical choices shaped the survivorship experience. The aim was to advance personalized, patient-centered care. This is accomplished by providing clinicians and patients with robust, evidence-based insights into recovery trajectories and long-term well-being. The results may also inform the wider research community by pinpointing key periods for supportive interventions and shaping future psycho-oncology and survivorship studies.

## 1. Introduction

Breast cancer (BC) remains the most commonly diagnosed malignancy and the leading cause of cancer-related mortality among women worldwide, with an estimated 2.3 million new cases annually [1]. While advances in early detection and multimodal treatment have significantly improved survival rates, increasingly the literature recognizes that survival alone is an insufficient measure of therapeutic success. Increasing emphasis is being placed on long-term survivorship outcomes, particularly QoL, which encompasses physical, emotional, social, and functional well-being [2,3]. Surgical treatment constitutes a pivotal stage in the BC trajectory, with its implications for QoL more and more recognized as a critical focus of clinical and psychosocial research [4].

A wide spectrum of surgical options is now available for BC patients, including BCS, MA and with immediate or delayed breast reconstruction. These approaches differ substantially not only in clinical complexity and recovery timelines but also in their psychosocial consequences [5]. BCS is often associated with more favorable QoL outcomes, particularly in terms of body image, psychological well-being, and sexual health [6]. Conversely, mastectomy, especially when performed without reconstruction, has been linked to adverse emotional and social outcomes, including depression, reduced self-esteem, and social withdrawal, whereas breast reconstruction appears to mitigate these adverse effects [7,8]. While reconstructive surgery may attenuate some negative sequelae of mastectomy, findings remain inconsistent, with some studies reporting sustained improvements in QoL and others noting delayed or incomplete recovery [9,10].

Quality-of-life trajectories are dynamic rather than static, evolving over the course of survivorship. These patterns are influenced by both demographic characteristics and treatment-related factors. Age significantly influences post-surgical QoL, with younger survivors reporting greater psychological and functional challenges—including body image concerns, anxiety, fatigue, and sexual dysfunction—compared with older survivors and age-matched controls [10,11]. Recent longitudinal analyses suggest that QoL may follow a non-linear, or curvilinear, pattern over time, characterized by early post-treatment gains followed by stabilization or even decline in later survivorship phases [12].

Despite the wealth of studies evaluating QoL after BC surgery, existing evidence remains heterogeneous, and comparative analyses across surgical modalities and age groups are limited. A more nuanced understanding is needed of how different surgical approaches and patient characteristics interact to shape long-term quality-of-life outcomes. Meta-regression techniques, which allow for the synthesis of effect sizes while accounting for covariates such as age and time since surgery, offer a powerful methodological approach to address these knowledge gaps [13]. The present meta-analysis aims to evaluate the dynamic trajectories of global QoL following BC surgery, with particular focus on the influence of surgical type and patient age. To reach the aim we formulated the following objectives: (1) conduct subgroup analyses to explore differences in global QoL outcomes across distinct patient subgroups; (2) model the longitudinal trajectory of global QoL in BC patients following surgical treatment, capturing patterns of change over time; (3) estimate and compare trajectory models of global QoL according to different surgical modalities and age groups; (4) perform meta-regression analyses to identify and quantify the effects of clinical and demographic moderators on global QoL outcomes after BC surgery.

By integrating data across multiple studies and modeling curvilinear trends over time, this study sought to inform more individualized survivorship care planning and support shared decision-making in surgical consultations. Understanding these temporal patterns allows clinicians to anticipate periods of heightened vulnerability or recovery and to adjust interventions accordingly. Such evidence-based insights can guide patients and providers in selecting surgical options that align not only with clinical outcomes but also with long-term quality-of-life priorities.

## 2. Materials and Methods

Study Design and Eligibility Criteria: This systematic review and meta-analysis was conducted in accordance with PRISMA guidelines [14]. We conducted a comprehensive literature search across the biomedical databases Scopus, CINAHL, Embase, APA PsycArticles, PubMed, SciELO, LILACS, and Global Index Medicus. The search strategy is provided in Supplementary Table S3. We included original studies if they reported QoL outcomes in BC patients following surgical treatment, used validated instruments such as the EORTC QLQ-C30 or BREAST-Q, and provided sufficient statistical information (means, standard deviations, and sample sizes) to estimate effect sizes at multiple post-surgical time points. We included only papers published between 2000 and 2024. We excluded studies that used alternative instruments to maintain methodological consistency. We used the Covidence online systematic review platform to manage study selection [15]. After uploading the retrieved publications for automatic deduplication, reviewers [I.M., M.E., I.M.A.M., F.T., J.A.] independently screened titles and abstracts against the inclusion and exclusion criteria. When reviewers disagreed on a study’s eligibility, they resolved the issue through discussion with the principal investigator. We applied the same procedure to full-text screening. We recorded reasons for article exclusion and summarized them in a PRISMA flow diagram (Figure S1, Supplementary Materials). Reviewers [I.M., M.E., I.M.A.M., F.T., J.A.] independently extracted data using a customized template, capturing study characteristics, methodological details, and key findings. We conducted a critical appraisal of each included study to evaluate methodological rigor and risk of bias using the Joanna Briggs Institute (JBI) critical appraisal tool [16,17] and calculated the effect size for each study to allow for standardized comparison (Tables S1 and S2, Supplementary Materials).

Data Collection and Preparation: A total of 123 observations from 45 studies were included [18,19,20,21,22,23,24,25,26,27,28,29,30,31,32,33,34,35,36,37,38,39,40,41,42,43,44,45,46,47,48,49,50,51,52,53,54,55,56,57,58,59,60,61,62]. Extracted variables included: study identifiers, patient age categories (<45, 45–60, >60 years), surgical modality (MA, BCS, Mx+IR, Other), instrument used, time since surgery, mean global QoL scores, standard deviations, and sample sizes. We analyzed all forms of immediate reconstruction following mastectomy collectively under the “Mx+IR” domain, as limited reporting and heterogeneity across studies precluded stratification by reconstruction type, while acknowledging that implant-based and autologous approaches may differentially influence QoL outcomes. We categorized postoperative time points as 0–6 months (coded as 3), 7–15 months (12), 16–30 months (24), 31–54 months (48), 55–72 months (60), and >73 months (120). We retained original time values when they were reported. Data Analysis: All statistical analyses were performed using R v 4.4.1. software [63]. For the statistical analysis, we applied multilevel random-effects meta-analysis models to account for both between-study and within-study variance. All global QoL scores were presented on a 0–100 scale. Trajectory modeling included linear, quadratic, and logarithmic functional forms. Model fit was evaluated using Akaike Information Criterion (AIC) and Bayesian Information Criterion (BIC) [64].

Global QoL trajectories were classified as stable high, stable moderate, persistently low, improving, declining, U-shaped, or inverted-U based on regression coefficients. Effect sizes were calculated as raw mean differences, with variances and confidence intervals derived using standard meta-analytic procedures. The variance for each effect size was computed as the square of the harmonized score’s standard deviation divided by the sample size. Standard errors and 95% confidence intervals were calculated using established meta-analytic procedures for raw mean differences. Between-study heterogeneity was quantified using the following: (1) Cochran’s Q statistic: Testing the null hypothesis of homogeneity [65]. (2) I^2^ statistic: Proportion of total variation due to heterogeneity [66]. (3), τ^2^ (tau-squared): Estimate of between-study variance. Values of I^2^ around 25%, 50%, and 75% were interpreted as low, moderate, and high heterogeneity, respectively [67].

Subgroup and Meta-Regression Analyses: Subgroup analyses explored global QoL trajectories by surgical type, age group, and time interval. Meta-regression models tested time as a continuous moderator and examined interactions between surgery type and age group. Statistical significance was set at α = 0.05. Bias and Sensitivity Analyses: Publication bias was evaluated using funnel plots and Egger’s regression test [68]. Leave-one-out sensitivity analysis was conducted to confirm the robustness of findings. Visualization: We constructed forest plots and trajectory curves to illustrate global QoL patterns across time points, surgical modalities, and age groups. We applied weighting to individual study estimates and reported them with corresponding 95% confidence intervals. Ethical Considerations and Data Availability: As this study did not directly involve human participants or animals, ethical approval was not required. All materials, data extraction templates, analysis code, and protocols are available from the corresponding author upon reasonable request. Generative AI tools were not used in data extraction or analysis.

## 3. Results

### 3.1. Global Quality of Life

#### 3.1.1. Descriptive Statistics

A total of 123 observations from 45 studies were included. Most assessments used the EORTC QLQ-C30 (89.4%), with fewer using BREAST-Q (10.6%). The most common surgery types were mastectomy alone (34.1%) and breast-conserving surgery (31.7%). Time since surgery was mainly within 0–15 months (57%), and the majority of patients were aged 45–60 years (68.3%).

#### 3.1.2. Subgroup Analysis of Global QoL

**Figure 1:**
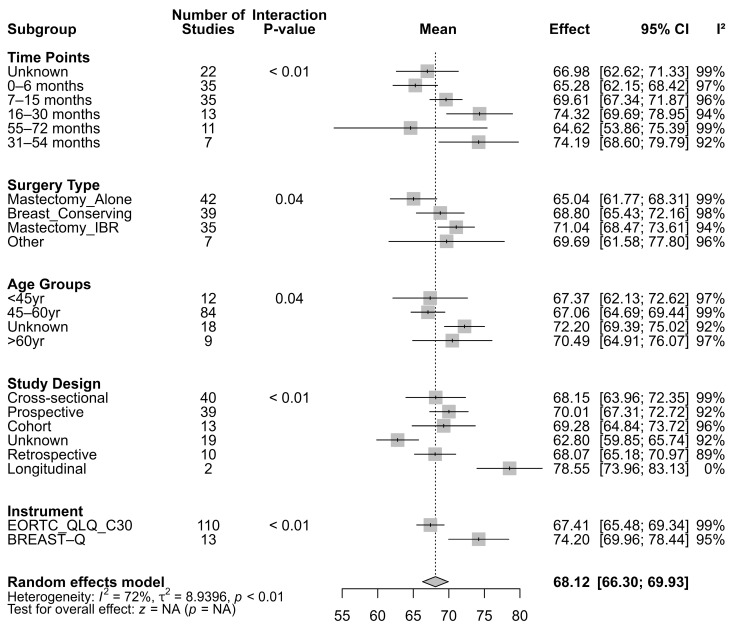
Pooled global QoL scores are harmonized on a 0–100 scale; higher scores indicate better quality of life. Random-effects models were used with REML estimation. I^2^ reflects the proportion of variance due to heterogeneity. *p* values indicate whether subgroup differences significantly explain heterogeneity.

**Figure A1:**
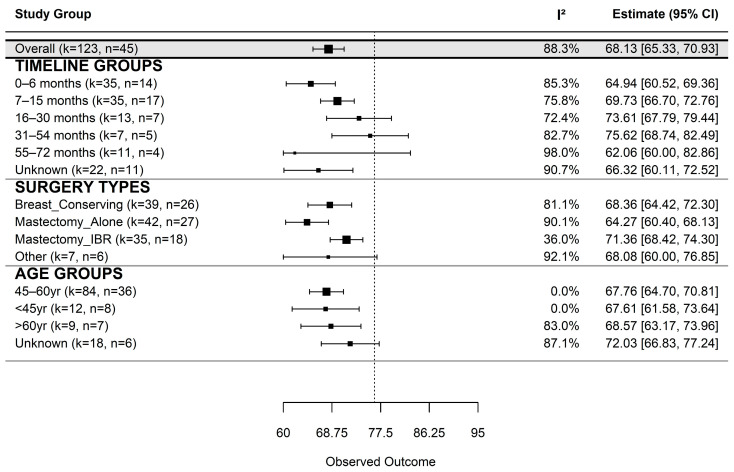
Forest Plot of Global QoL by Timeline, Surgery Type, and Age Group.

The subgroup meta-analysis revealed significant variation in global QoL following BC surgery across time points, surgical types, age groups, study designs, and measurement instruments. The overall pooled estimate of global QoL was 68.13 (SE = 1.43, 95% CI: 65.33–70.93, *p* < 0.001), with high heterogeneity (I^2^ = 88.28%) (Figure 1; Figure A1 and Table A1, Appendix A). When stratified by time since surgery, QoL was lowest in the 0–6 month subgroup (64.94, SE = 2.26, 95% CI: 60.52–69.36, *p* < 0.001), gradually increased over time, and peaked at 31–54 months (75.62, SE = 3.51, 95% CI: 68.74–82.49, *p* < 0.001), before showing a decline at 55–72 months (62.06, SE = 10.61, 95% CI: 41.26–82.86, *p* < 0.001).

By surgery type, the highest pooled QoL was observed for Mx+IR (71.36, SE = 1.50, 95% CI: 68.42–74.30, *p* < 0.001), followed by BCS (68.36, SE = 2.01, 95% CI: 64.42–72.30, *p* < 0.001), and Other procedures (68.08, SE = 4.47, 95% CI: 59.32–76.85, *p* < 0.001). Women undergoing MA reported the lowest QoL (64.27, SE = 1.97, 95% CI: 60.40–68.13, *p* < 0.001), with this group also showing high heterogeneity (I^2^ = 90.07%).

In terms of age, the 45–60 year group had a pooled estimate of 67.76 (SE = 1.56, 95% CI: 64.71–70.81, *p* < 0.001). The <45 year group showed a similar estimate (67.61, SE = 3.08, 95% CI: 61.58–73.64, *p* < 0.001), while the >60 year group reported 68.57 (SE = 2.75, 95% CI: 63.17–73.96, *p* < 0.001). The unknown age category had the highest estimate (72.03, SE = 2.66, 95% CI: 66.83–77.24, *p* < 0.001).

#### 3.1.3. Trajectory Modelling for Global QoL

The trajectory analysis by time points revealed clear changes in global QoL over the post-surgical period among BC patients. The quadratic model provided the best fit for the QoL trajectory data, with the lowest AIC (972.18) and BIC (985.10) (Table A2, Appendix A). In contrast, the logarithmic model showed higher values (ΔAIC = 13.02, ΔBIC = 10.48), and the linear model performed worst (ΔAIC = 17.08, ΔBIC = 14.54). These differences confirm that the quadratic specification best captured the trajectory of QoL over time.

**Figure 2:**
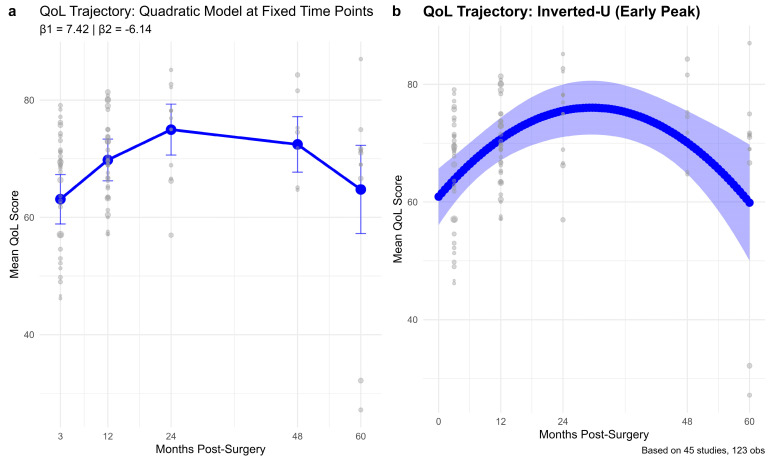
Trajectory of global QoL over 60 months following surgery.

The trajectory analysis by time points revealed clear changes in global QoL over the post-surgical period among breast cancer patients (Figure 2). Predicted QoL was lowest in the 0–6 month period (63.10, 95% CI: 58.90–67.31), reflecting the immediate post-surgical burden. Scores improved during the 7–15 month window (69.80, 95% CI: 66.25–73.34) and peaked at 16–30 months (74.97, 95% CI: 70.62–79.32). QoL then declined slightly at 31–54 months (72.45, 95% CI: 67.71–77.19) and more markedly by 55–72 months (64.76, 95% CI: 57.24–72.28). This trajectory is consistent with a quadratic pattern of recovery and decline over time.

#### 3.1.4. Trajectory Model Estimates for QoL by Surgical Modality

**Figure 3:**
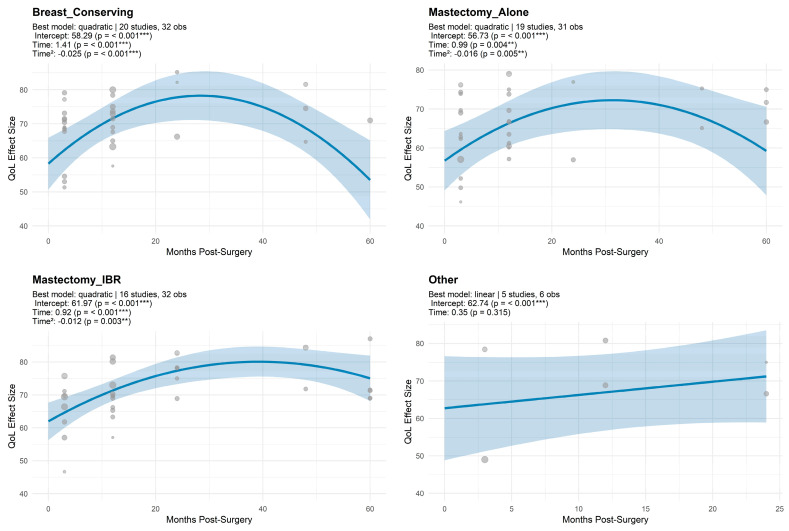
Unadjusted meta-regression of global QoL scores over time by surgery. *** indicates *p* < 0.001; ** indicates *p* < 0.01.

Quadratic trajectory models were successfully fitted for the three main surgical subgroups. The BCS group (k = 32, 20 studies) showed the steepest rise (β = 1.41 ± 0.39, *p* < 0.001) with a mild quadratic deceleration (β = −0.02 ± 0.01, *p* < 0.001), and high residual heterogeneity (QE = 1180.85, *p* < 0.001) (Figure 3; Table A3, Appendix A). The MA group (k = 31, 19 studies) demonstrated a slower upward trend (β = 0.99 ± 0.34, *p* = 0.015) with a weaker quadratic effect (β = −0.02 ± 0.01, *p* = 0.015), alongside substantial heterogeneity (QE = 1714.69, *p* < 0.001). The Mx+IR group (k = 32, 16 studies) had significant increase in QoL over time (β = 0.92 ± 0.25, *p* < 0.001) with a modest flattening effect (β = −0.01 ± 0.00, *p* = 0.003), and moderate residual heterogeneity (QE = 371.04, *p* < 0.001). In contrast, the Other surgeries subgroup (k = 6, 5 studies) showed no significant linear trajectory (β = 0.35 ± 0.35, *p* = 0.315), and a quadratic model could not be fitted (QE = 124.30, *p* < 0.001).

#### 3.1.5. Trajectory Model Estimates for Global QoL by Age

**Figure 4:**
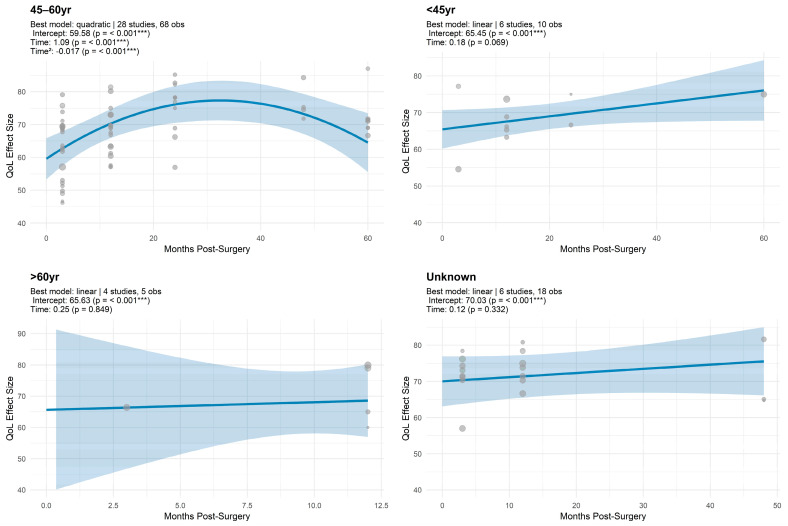
Unadjusted meta-regression of Global QoL scores over time by age group. *** indicates *p* < 0.001.

Age-stratified analyses demonstrated distinct patterns of QoL trajectories across groups. For the 45–60 year group (28 studies, 68 observations), QoL followed a significant quadratic trajectory, starting at 59.58 (*p* < 0.001), increasing over time (β = 1.09, *p* < 0.001), and then declining (quadratic term β = −0.02, *p* < 0.001) (Figure 4; Table A4, Appendix A). In the <45 year group (6 studies, 10 observations), the best-fitting model was linear, with an intercept of 65.45 (*p* < 0.001) and a positive but borderline non-significant slope (β = 0.18, *p* = 0.069). For the >60 year group (4 studies, 5 observations), the model was also linear, starting higher at 65.63 (*p* < 0.001), with a flat trajectory over time (β = 0.25, *p* = 0.849). In the Unknown age group (6 studies, 18 observations), the intercept was 70.03 (*p* < 0.001), with a non-significant upward slope (β = 0.12, *p* = 0.332).

#### 3.1.6. Meta Regression for Global QoL

Multivariate meta-regression examined the effects of time, surgical type, and age on QoL trajectories. Findings confirmed a significant curvilinear effect of time, with a positive linear slope (β = 7.30, *p* = 0.033) and a negative quadratic term (β = −5.55, *p* = 0.032), indicating an initial increase in QoL followed by flattening and decline. Compared to BCS (reference), MA was associated with significantly lower scores (β = −6.21, *p* < 0.001), while Mx+IR showed a significant decrease (β = −4.57, *p* = 0.027) and Other procedures were non-significant (β = −1.29, *p* = 0.709) (Table 1).

Interaction terms showed that MA had a significantly steeper negative time slope (β = −3.19, *p* = 0.005) with a compensatory positive quadratic effect (β = 4.08, *p* < 0.001). Other surgery showed a strong negative time interaction (β = −27.82, *p* = 0.049) with an additional negative quadratic effect (β = −34.72, *p* = 0.025). For age, no significant main effects were observed, although the >60 year group displayed a significant positive time interaction (β = 19.97, *p* = 0.008), suggesting greater improvement over time compared to the reference.

The omnibus test of moderators showed that time points (χ^2^ = 25.61, df = 4, *p* < 0.0001), surgery types (χ^2^ = 40.53, df = 3, *p* < 0.0001), age groups (χ^2^ = 67.09, df = 3, *p* < 0.0001), and the instrument used (χ^2^ = 4.07, df = 1, *p* = 0.044) significantly explained heterogeneity in overall QoL scores. In contrast, study design was not significant (χ^2^ = 3.73, df = 4, *p* = 0.445) (Table A5, Appendix A).

**Figure A2:**
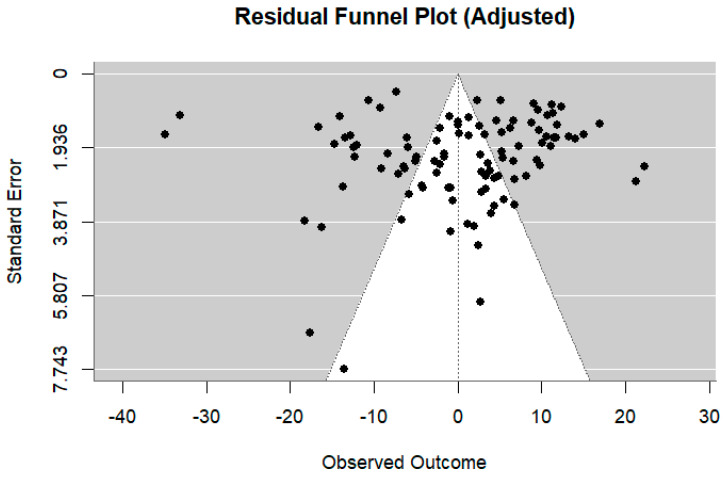
Funnel Plot (Adjusted).

Egger’s regression test showed no evidence of small-study effects. The intercept was non-significant (β = −0.93, SE = 2.06, *p* = 0.652), and the regression slope of residuals on precision was also non-significant (β = 2.10, SE = 3.11, *p* = 0.502). The overall model explained minimal variance (R^2^ = 0.005, adjusted R^2^ = −0.005), with an F-statistic of 0.45 (*p* = 0.502), indicating a low likelihood of publication bias (Figure A2, Appendix A).

## 4. Discussion

This study provides a comprehensive synthesis of global QoL trajectories following different surgical treatments for BC, highlighting both the time-dependent complexity and the differential impact of surgical modality and patient age. The observed curvilinear trends—early postoperative improvements peaking between 31 and 54 months, followed by stabilization or decline—align with prior longitudinal studies demonstrating that QoL evolves dynamically throughout survivorship [2,11,69,70,71]. These temporal trajectories are likely shaped by the dynamic interplay of physical recovery, psychosocial adjustment, and long-term survivorship challenges. In the immediate postoperative period, acute functional limitations, treatment-related toxicities, and heightened emotional distress contribute to reduced QoL. With time, progressive physical recovery, psychological adaptation, and reintegration into social and occupational roles facilitate gradual improvement, culminating in peak QoL between 31 and 54 months after surgery. The subsequent plateau or modest decline likely reflects the persistence or emergence of late effects—such as chronic pain, lymphedema, and body image disturbance—together with enduring psychosocial stressors, including fear of recurrence, relational or occupational difficulties, and age-related comorbidities.

The present analyses revealed substantial heterogeneity across studies, with several comparisons exhibiting I^2^ values above 80%. This variability likely reflects differences in study populations and treatment protocols. Contributing factors may include variation in adjuvant therapies—such as chemotherapy, radiotherapy, endocrine therapy, and targeted biologics—which can differentially influence symptom burden, functional recovery, and long-term quality of life. Cultural and healthcare system differences may affect patient-reported outcomes, as societal norms surrounding body image, emotional expression, and family support shape perceived well-being. At the same time, disparities in healthcare infrastructure, access to specialized services, and follow-up protocols can alter recovery trajectories. Socioeconomic determinants—including income, education, employment, and social support—also modulate both rehabilitation resources and the psychological resilience required for postoperative adjustment. Although this heterogeneity does not negate the observed trends, it calls for cautious interpretation of pooled estimates and highlights the importance of considering both contextual and patient-level factors when applying these findings clinically.

Surgical modality emerged as a pivotal moderator of global QoL trajectories. BCS consistently demonstrated favorable outcomes, corroborating prior studies showing higher overall satisfaction, preserved functional capacity, and reduced symptom burden compared to mastectomy [19,21,23,33,52].

Acil et al. [19] reported superior functional and symptom scores among BCS patients, with younger patients showing a trend toward better recovery. Similarly, Cortés-Flores et al. [22] highlighted stronger early QoL gains and better body image among BCS patients. BCS exhibited an initial rate of QoL improvement comparable to Mx+IR, with some domains plateauing over time, potentially reflecting the impact of adjuvant therapies and long-term sequelae [72]. Mx+IR was associated with most sustained QoL over time, aligning with literature emphasizing psychosocial benefits and patient satisfaction [24,33,34]. Nevertheless, reconstruction does not universally guarantee superior outcomes, as postoperative expectations, complications, and individual psychosocial factors mediate long-term QoL [22,24,31]. Elevated pain and functional burdens observed in reconstruction cohorts suggest early gains may be tempered by physical and emotional demands.

In contrast, MA consistently corresponded with the lowest QoL scores and the slowest recovery trajectories, echoing findings from literature [18,31,34]. Patients with MA frequently experience poorer long-term well-being, elevated psychosocial burden, compromised body image, and diminished sexual function [18,20,22,34]. Delayed reconstruction further compounds these challenges, highlighting the need for targeted supportive interventions.

Cohen et al. [27] examined long-term psychological adjustment and reported that although BCS is associated with favorable early QoL, psychological distress may increase around 40 months post-surgery, likely reflecting recurrence anxiety and evolving body image concerns. Similarly, Han et al. [57] observed early QoL improvements following BCT, with BCT patients reporting better body image and satisfaction; however, patients undergoing reconstruction experienced financial and future-related concerns, potentially explaining gradual declines in QoL over time. Gillies et al. [41] further emphasized the role of psychosocial and behavioral factors—including emotional distress, fear of recurrence, and social support—in shaping QoL. Younger age was consistently associated with better QoL, aligning with evidence on age-moderated recovery trajectories. Collectively, these findings highlight that QoL outcomes are influenced not only by surgical choice but also by individual psychosocial and demographic factors, reinforcing the need for personalized survivorship care.

Kim et al. [44] demonstrated that perceived cosmetic outcomes significantly affect QoL, particularly body image, sexual well-being, and psychosocial adjustment. Lagendijk et al. [43,46] underscored the importance of patient-reported outcome measures (PROMs) in capturing aspects of recovery not reflected in clinician-reported outcomes, supporting longitudinal QoL monitoring. Additional evidence indicates that BCS generally yields superior QoL, although adjuvant therapies and healthcare context modulate recovery [36,38]. Qin et al. [46] and Shi et al. [48] demonstrated that reconstruction offers psychosocial benefits, albeit with delayed recovery, highlighting the interplay between short-term burden and long-term QoL gains.

Notably, Szutowicz-Wydra et al. [62] found that Polish patients undergoing mastectomy with reconstruction or BCT reported comparable overall QoL, suggesting that in certain cultural and healthcare contexts, psychosocial adaptation, expectations, and support may mitigate differences between surgical types. These findings collectively indicate that while surgical modality is an important determinant of QoL, psychosocial, cultural, and individual factors can equalize outcomes, underscoring the necessity of tailored care strategies.

Age did not independently predict overall QoL but significantly moderated recovery trajectories. Women aged 45–60 exhibited initial improvements followed by decline, consistent with mid-life stressors. Older patients (>60 years) experienced more gradual improvements, likely influenced by slower functional recovery and comorbidities, whereas younger patients (<45 years) demonstrated more rapid and sustained recovery, in line with evidence on resilience and adaptive coping [19,32,34,71].

Taken together, these findings suggest that global QoL recovery after breast cancer surgery is shaped by surgical modality, patient age, psychosocial adaptation, and cultural context. When clinically feasible, BCS should be prioritized, while mastectomy patients—particularly those without reconstruction—may benefit from early psychosocial, functional, and sexual health interventions. Routine assessment of global QoL through PROMs is essential for shared decision-making, expectation management, and timely referral to multidisciplinary support services.

### 4.1. Strengths and Limitations

This synthesis benefits from advanced meta-regression techniques capturing nonlinear QoL trajectories, a large aggregated sample, and detailed subgroup analyses. Residual heterogeneity suggests unmeasured confounders, such as socioeconomic status, adjuvant treatments, and cultural factors, may impact outcomes. Scarcity of data in older adults and less common procedures limits precision and generalizability.

### 4.2. Future Directions

Future research should integrate longitudinal, patient-centered designs, consider surgical type, age, psychosocial factors, and cultural context and assess preoperative expectations, decisional regret, and culturally tailored survivorship interventions to optimize outcomes for diverse BC populations. In addition, greater attention should be given to clinical determinants—such as cancer grade, lymph node involvement, and molecular subtype—to better understand their impact on postoperative quality of life trajectories. Addressing these factors may help explain the heterogeneity observed across studies and support the advancement of more personalized survivorship care models.

## 5. Conclusions

This meta-regression analysis reveals that global QoL following BC surgery follows a distinct curvilinear trajectory, characterized by an initial postoperative decline, progressive improvement peaking between 31 and 54 months, and a subsequent modest plateau. Among the surgical approaches examined, BCS was associated with the most favorable and sustained QoL improvements over time. In contrast, MA demonstrated the least favorable trajectory, with significantly lower QoL scores and a slower recovery process. Mx+IR showed intermediate outcomes, exhibiting a smaller and borderline-significant reduction in QoL relative to BCS.

Age-related effects were generally limited, with only patients aged 45–60 years exhibiting a significant trajectory marked by early QoL gains followed by decline; younger and older cohorts showed no significant temporal changes, although younger (<45 years) and older (>60 years) patients showed relatively stable, linear trajectories over time. High residual heterogeneity across analyses highlights substantial variability in individual patient experiences, underscoring the complexity of QoL trajectories in this population.

For patients considering MA or Mx+IR, it is essential to communicate the increased risk of prolonged psychosocial burden and to proactively emphasize the importance of early psychosocial interventions, structured education, and ongoing supportive care. Patients aged 45–60 years warrant particular attention, as their QoL trajectories reveal initial improvement followed by decline, underscoring the need for sustained monitoring and tailored psychosocial support during this critical period. By contrast, younger (<45 years) and older (>60 years) patients tend to demonstrate more stable trajectories, though their distinct psychosocial and functional needs should still be addressed through age-appropriate interventions. The incorporation of PROMs into surgical consultations should be routine practice, as these tools can facilitate shared decision-making, align surgical choices with patient preferences and values, and provide a framework for ongoing evaluation of recovery trajectories. Embedding PROMs into the consultation process ensures that treatment decisions are informed not only by oncologic safety and reconstructive feasibility but also by robust, evidence-based projections of long-term well-being.

The delineated curvilinear trajectories of QoL provide critical guidance for individualized survivorship planning and follow-up scheduling. Patients undergoing BCS may be adequately monitored through standard oncologic follow-up intervals, with targeted assessments reserved for late-emerging psychosocial or functional concerns. In contrast, patients receiving MA can benefit from enhanced and prolonged follow-up, including structured psychosocial evaluations at shorter intervals and early integration of supportive care interventions. Those undergoing Mx+IR may benefit from personalized follow-up strategies that simultaneously address oncologic surveillance, functional rehabilitation, and adaptation to reconstructed body image. Integrating these trajectory-informed insights into survivorship care enables clinicians to optimize follow-up schedules, allocate supportive care resources efficiently, and proactively mitigate potential declines in QoL, thereby promoting sustained well-being and patient-centered outcomes.

Overall, these results highlight the dual importance of surgical modality and patient age in shaping postoperative QoL. They also emphasize the clinical responsibility to move beyond survival-focused counseling and incorporate anticipated QoL outcomes into treatment planning. Doing so supports informed decision-making, empowers patients, and ensures that care pathways are aligned with both medical and psychosocial dimensions of survivorship.

## Figures and Tables

**Table 1:** Multivariate Meta-Regression Model for Global QoL Trajectories by Age and Surgery Type.

Predictor	Estimate	SE	z-Value	*p*-Value	95% CI (LB–UB)
Intercept	77.49	3.41	22.73	<0.001	70.81–84.17
Time (linear)	7.30	3.43	2.13	0.033	0.58–14.02
Time^2^ (quadratic)	−5.55	2.59	−2.14	0.032	−10.63–−0.47
Age > 60 yr	5.35	5.01	1.07	0.286	−4.47–15.17
Age 45–60 yr	1.42	2.68	0.53	0.596	−3.83–6.66
Age Unknown	−4.20	5.75	−0.73	0.465	−15.46–7.06
Mastectomy Alone	−6.21	0.87	−7.11	<0.001	−7.92–−4.50
Mastectomy with IBR	−4.57	2.06	−2.22	0.027	−8.61–−0.53
Other surgery	−1.29	3.45	−0.37	0.709	−8.06–5.48
Time × Age > 60 yr	19.97	7.48	2.67	0.008	5.31–34.63
Time × Age 45–60 yr	1.57	2.85	0.55	0.580	−4.00–7.15
Time × Age Unknown	1.79	4.32	0.41	0.679	−6.68–10.26
Time^2^ × Age 45–60 yr	−3.34	2.08	−1.61	0.108	−7.41–0.74
Time^2^ × Age Unknown	−2.54	4.77	−0.53	0.594	−11.89–6.81
Time × Mastectomy Alone	−3.19	1.13	−2.81	0.005	−5.41–−0.97
Time × Mastectomy with IBR	−2.14	2.49	−0.86	0.391	−7.02–2.75
Time × Other surgery	−27.82	14.11	−1.97	0.049	−55.47–−0.18
Time^2^ × Mastectomy Alone	4.08	0.83	4.95	<0.001	2.47–5.70
Time^2^ × Mastectomy with IBR	2.93	1.70	1.72	0.085	−0.40–6.27
Time^2^ × Other surgery	−34.72	15.50	−2.24	0.025	−65.10–−4.35

## Data Availability

Data supporting the results of this study are available from the corresponding author upon reasonable request.

## Supplementary Materials

The following supporting information can be downloaded at: https://www.mdpi.com/article/10.3390/cancers17193108/s1, Figure S1: title “PRISMA flow diagram”; Table S1: title “Effect size by study”; Table S2: title “Study rigor”; Table S3: title “Search strategy string”.

## Abbreviations

The following abbreviations are used in this manuscript:

AIC	Akaike information criterion
BC	breast cancer
BCS	breast-conserving surgery
BIC	Bayesian information criterion
IBR	immediate breast reconstruction
Mx+IR	mastectomy with immediate reconstruction
PROMs	patient-reported outcome measures
MA	Mastectomy Alone
QoL	quality of life

## Appendix A

**Table A1:** Subgroup analysis of Global Quality of Life (QoL) by Time Points Post-Surgery.

Group	k	n_Studies	Estimate	SE	95% CI (LB–UB)	τ^2^	I^2^ (%)	*p*-Value
Overall	123	45	68.13	1.43	65.33–70.93	47.63	88.28	<0.001
Time since surgery								
0–6 months	35	14	64.94	2.26	60.52–69.36	34.67	85.32	<0.001
7–15 months	35	17	69.73	1.55	66.70–72.76	19.21	75.78	<0.001
16–30 months	13	7	73.61	2.97	67.79–79.44	28.50	72.40	<0.001
31–54 months	7	5	75.62	3.51	68.74–82.49	28.64	82.73	<0.001
55–72 months	11	4	62.06	10.61	41.26–82.86	224.62	98.03	<0.001
Unknown	22	11	66.32	3.17	60.11–72.52	53.84	90.70	<0.001
Surgery type								
Breast-Conserving	39	26	68.36	2.01	64.42–72.30	19.67	81.10	<0.001
Mastectomy Alone	42	27	64.27	1.97	60.40–68.13	40.40	90.07	<0.001
Mastectomy IBR	35	18	71.36	1.50	68.42–74.30	5.56	36.02	<0.001
Other	7	6	68.08	4.47	59.32–76.85	111.06	92.09	<0.001
Age group								
45–60 yr	84	36	67.76	1.56	64.71–70.81	0.00	0.00	<0.001
<45 yr	12	8	67.61	3.08	61.58–73.64	0.00	0.00	<0.001
>60 yr	9	7	68.57	2.75	63.17–73.96	24.10	83.01	<0.001
Unknown	18	6	72.03	2.66	66.83–77.24	24.78	87.05	<0.001

Footnote: Mean QoL estimates are based on pooled scores using a 0–100 harmonized scale, where higher values indicate better emotional quality of life. Random-effects multilevel meta-analyses were conducted using REML estimation. I^2^ represents the proportion of total variability due to between-study heterogeneity. All *p*-values refer to the test of pooled effects within each subgroup.

**Table A2:** Overall trajectory modelling results for Global QoL.

Model	AIC	BIC	R^2^	ΔAIC	ΔBIC
Quadratic	972.18	985.10	1	0.00	0.00
Logarithmic	985.20	995.58	1	13.02	10.48
Linear	989.26	999.64	1	17.08	14.54

**Table A3:** Trajectory Model Estimates for Global Quality of Life (QoL) by Surgical Modality.

Surgery Type	k	Studies	Intercept	Linear Term	Quadratic Term	R^2^ (QM *p*-Value)	QE (*p*-Value)
Breast_Conserving	32	20	58.29 ± 3.88 ***	1.41 ± 0.39 ***	−0.02 ± 0.01 ***	(<0.001)	1180.85 (<0.001)
Mastectomy_Alone	31	19	56.73 ± 3.90 ***	0.99 ± 0.34 **	−0.02 ± 0.01 **	(0.015)	1714.69 (<0.001)
Mastectomy_IBR	32	16	61.97 ± 2.92 ***	0.92 ± 0.25 ***	−0.01 ± 0.00 **	(<0.001)	371.04 (<0.001)
Other	6	5	62.74 ± 7.10 ***	0.35 ± 0.35	—	(0.315)	124.30 (<0.001)

Notes: *** indicates *p* < 0.001; ** indicates *p* < 0.01.

**Table A4:** Trajectory Model Estimates for Global Quality of Life (QoL) by Age Group.

Age Group	k	Studies	Intercept	Linear Term	Quadratic Term	R^2^ (QM *p*-Value)	QE (*p*-Value)
45–60 yr	68	28	59.58 ± 3.20 ***	1.09 ± 0.29 ***	−0.02 ± 0.00 ***	(<0.001)	3180.58 (<0.001)
<45 yr	10	6	65.45 ± 2.68 ***	0.18 ± 0.10	—	(0.069)	101.86 (<0.001)
>60 yr	5	4	65.63 ± 13.48 ***	0.25 ± 1.29	—	(0.849)	75.31 (<0.001)
Unknown	18	6	70.03 ± 3.53 ***	0.12 ± 0.12	—	(0.332)	212.75 (<0.001)

Notes: *** *p* < 0.001.

**Table A5:** Omnibus Test of Moderators: Overall QoL.

Moderator	Chi-Square	df	*p*-Value
Time Points	25.61	4	<0.0001
Surgery Types	40.53	3	<0.0001
Age Groups	67.09	3	<0.0001
Instrument Used	4.07	1	0.044
Study Design	3.73	4	0.445

## References

[1] Sung H., Ferlay J., Siegel R.L., Laversanne M., Soerjomataram I., Jemal A., Bray F. (2021). Global Cancer Statistics 2020: GLOBOCAN Estimates of Incidence and Mortality Worldwide for 36 Cancers in 185 Countries. CA Cancer J. Clin..

[2] Montazeri A. (2008). Health-related quality of life in breast cancer patients: A bibliographic review of the literature from 1974 to 2007. J. Exp. Clin. Cancer Res..

[3] Deshpande P.R., Rajan S., Sudeepthi B.L., Abdul Nazir C.P. (2011). Patient-reported outcomes: A new era in clinical research. Perspect. Clin. Res..

[4] Al-Ghazal S., Fallowfield L., Blamey R. (2000). Comparison of psychological aspects and patient satisfaction following breast conserving surgery, simple mastectomy and breast reconstruction. Eur. J. Cancer.

[5] Metcalfe K.A., Semple J., Quan M.-L., Vadaparampil S.T., Holloway C., Brown M., Bower B., Sun P., Narod S.A. (2011). Changes in Psychosocial Functioning 1 Year After Mastectomy Alone, Delayed Breast Reconstruction, or Immediate Breast Reconstruction. Ann. Surg. Oncol..

[6] Parker P.A., Youssef A., Walker S., Basen-Engquist K., Cohen L., Gritz E.R., Wei Q.X., Robb G.L. (2007). Short-Term and Long-Term Psychosocial Adjustment and Quality of Life in Women Undergoing Different Surgical Procedures for Breast Cancer. Ann. Surg. Oncol..

[7] Sisco M., Johnson D.B., Wang C., Rasinski K., Rundell V.L., Yao K.A. (2015). The quality-of-life benefits of breast reconstruction do not diminish with age. J. Surg. Oncol..

[8] Nano M.T., Gill P.G., Kollias J., Bochner M.A., Malycha P., Winefield H.R. (2005). Psychological impact and cosmetic outcome of surgical breast cancer strategies. ANZ J. Surg..

[9] Brandberg Y., Malm M., Blomqvist L. (2000). A Prospective and Randomized Study, “SVEA,” Comparing Effects of Three Methods for Delayed Breast Reconstruction on Quality of Life, Patient-Defined Problem Areas of Life, and Cosmetic Result. Plast. Reconstr. Surg..

[10] Avis N.E., Levine B., Naughton M.J., Case L.D., Naftalis E., Van Zee K.J. (2013). Age-related longitudinal changes in depressive symptoms following breast cancer diagnosis and treatment. Breast Cancer Res. Treat..

[11] Champion V.L., Wagner L.I., Monahan P.O., Daggy J., Smith L., Cohee A., Ziner K.W., Haase J.E., Miller K.D., Pradhan K. (2014). Comparison of younger and older breast cancer survivors and age-matched controls on specific and overall quality of life domains. Cancer.

[12] Pat-Horenczyk R., Kelada L., Kolokotroni E., Stamatakos G., Dahabre R., Bentley G., Perry S., Karademas E.C., Simos P., Poikonen-Saksela P. (2023). Trajectories of Quality of Life among an International Sample of Women during the First Year after the Diagnosis of Early Breast Cancer: A Latent Growth Curve Analysis. Cancers.

[13] Thompson S.G., Higgins J.P.T. (2002). How should meta-regression analyses be undertaken and interpreted?. Stat. Med..

[14] Page M.J., McKenzie J.E., Bossuyt P.M., Boutron I., Hoffmann T.C., Mulrow C.D., Shamseer L., Tetzlaff J.M., Akl E.A., Brennan S.E. (2021). The PRISMA 2020 statement: An updated guideline for reporting systematic reviews. BMJ.

[15] Covidence Systematic Review Software Veritas Health Innovation: Melbourne, Australia. www.covidence.org.

[16] Barker T.H., Hasanoff S., Aromataris E., Stone J.C., Leonardi-Bee J., Sears K., Habibi N., Klugar M., Tufanaru C., Moola S. (2025). The Revised JBI Critical Appraisal Tool for the Assessment of Risk of Bias for Cohort Studies. JBI Evid. Synth..

[17] Barker T.H., Hasanoff S., Aromataris E., Stone J.C., Leonardi-Bee J., Sears K., Klugar M., Tufanaru C., Moola S., Liu X.-L. (2025). The Revised JBI Critical Appraisal Tool for the Assessment of Risk of Bias for Analytical Cross-Sectional Studies. JBI Evid. Synth..

[18] Abebe E., Demilie K., Lemmu B., Abebe K. (2020). Female Breast Cancer Patients, Mastectomy-Related Quality of Life: Experience from Ethiopia. Int. J. Breast Cancer.

[19] Acil H., Cavdar I. (2014). Comparison of Quality of Life of Turkish Breast Cancer Patients Receiving Breast Conserving Surgery or Modified Radical Mastectomy. Asian Pac. J. Cancer Prev..

[20] Aerts L., Christiaens M., Enzlin P., Neven P., Amant F. (2014). Sexual functioning in women after mastectomy versus breast conserving therapy for early-stage breast cancer: A prospective controlled study. Breast.

[21] Cherian K., Acharya N.R., Bhargavan R.V., Augustine P., Krishnan J.K. (2022). Quality of Life Post Breast Cancer Surgery: Comparison of Breast Conservation Surgery versus Modified Radical Mastectomy in a Developing Country. South Asian J. Cancer.

[22] Cortés-Flores A.O., Morgan-Villela G., del Valle C.J.Z.-F., Jiménez-Tornero J., Juárez-Uzeta E., Urias-Valdez D.P., Garcia-González L.-A., Fuentes-Orozco C., Chávez-Tostado M., Macías-Amezcua M.D. (2014). Quality of Life Among Women Treated for Breast Cancer: A Survey of Three Procedures in Mexico. Aesthetic Plast. Surg..

[23] Dahlui M., Azzani M., Taib N.A., Hoong S.M., Jamaris S., Islam T. (2023). Breast conserving surgery versus mastectomy: The effect of surgery on quality of life in breast cancer survivors in Malaysia. BMC Women’s Health.

[24] Harcourt D.M., Rumsey N.J., Ambler N.R., Cawthorn S.J., Reid C.D., Maddox P.R., Kenealy J.M., Rainsbury R.M., Umpleby H.C. (2003). The Psychological Effect of Mastectomy with or without Breast Reconstruction: A Prospective, Multicenter Study. Plast. Reconstr. Surg..

[25] Nowicki A., Licznerska B., Rhone P. (2015). Evaluation of the quality of life of women treated due to breast cancer using amputation or breast conserving surgery in the early postoperative period. Pol. J. Surg..

[26] Songtish D., Akranurakkul P. (2021). Body Image and Quality of Life Correlation after Treatment in Thai Breast Cancer Patients. J. Med. Assoc. Thail..

[27] Cohen L., Hack T.F., de Moor C., Katz J., Goss P.E. (2000). The Effects of Type of Surgery and Time on Psychological Adjustment in Women After Breast Cancer Treatment. Ann. Surg. Oncol..

[28] Domenici L., Caputo G.G., Losco L., Di Taranto G., Lo Torto F., Pierazzi D.M., Governa M., Panici P.B., Ribuffo D., Cigna E. (2022). Muscle-Sparing Skin-Reducing Breast Reconstruction with Pre-Pectoral Implants in Breast Cancer Patients: Long-Term Assessment of Patients’ Satisfaction and Quality of Life. J. Investig. Surg..

[29] Hejl L., Raft J., Leufflen L., Rauch P., Buhler J., Abel-Decollogne F., Routiot T., Hotton J., Salleron J., Marchal F. (2020). Quality of life, anxiety, and postoperative complications of patients undergoing breast cancer surgery as ambulatory surgery compared to non-ambulatory surgery: A prospective non-randomized study. J. Gynecol. Obstet. Hum. Reprod..

[30] Hadi N., Soltanipour S., Talei A. (2012). Impact of modified radical mastectomy on health-related quality of life in women with early stage breast cancer. Arch. Iran. Med..

[31] Hassan B.A.R., Mohammed A.H., Al Zobair A.A., Wayyes A.M., Al-Jawadi H.K., Alsammarraie A.Z.A., Alabboodi M.K., Jiang J.X. (2024). Enhancing Women’s Quality of Life: Exploring the Impact of Mastectomy with and without Breast Reconstruction among Breast Cancer Survivors in Iraq. Asian Pac. J. Cancer Prev..

[32] Jayasinghe R., Fernando A., Jayarajah U., Seneviratne S. (2021). Post treatment quality of life among Sri Lankan women with breast cancer. BMC Cancer.

[33] Konieczny M., Fal A. (2023). The Influence of the Surgical Treatment Method on the Quality of Life of Women With Breast Cancer. Eur. J. Breast Health.

[34] Kouwenberg C.A.E.M., de Ligt K.M., Kranenburg L.W., Rakhorst H.M., de Leeuw D.M., Siesling S., Busschbach J.J., Mureau M.A.M.M. (2020). Long-Term Health-Related Quality of Life after Four Common Surgical Treatment Options for Breast Cancer and the Effect of Complications: A Retrospective Patient-Reported Survey among 1871 Patients. Plast. Reconstr. Surg..

[35] Moro-Valdezate D., Buch-Villa E., Peiró S., Morales-Monsalve M.D., Caballero-Gárate A., Martínez-Agulló Á., Checa-Ayet F., Ortega-Serrano J. (2012). Factors associated with health-related quality of life in a cohort of Spanish breast cancer patients. Breast Cancer.

[36] Nsaful J., Nartey E.T., Dedey F., Bediako-Bowan A., Appiah-Danquah R., Darko K., Ankrah L.N.A., Akli-Nartey C., Annan J.Y., Dei-Asamoa J. (2024). Quality of Life after Mastectomy with or without Breast Reconstruction and Breast-Conserving Surgery in Breast Cancer Survivors: A Cross-Sectional Study at a Tertiary Hospital in Ghana. Curr. Oncol..

[37] Pačarić S., Kristek J., Mirat J., Kondža G., Turk T., Farčić N., Orkić Ž., Nemčić A. (2018). The quality of life of Croatian women after mastectomy: A cross-sectional single-center study. BMC Public Health.

[38] Camejo N., Amarillo D., Castillo C., Guerrina M., Savio F., Carrasco M., Strazzarino N., Hernandez A.L., Herrera G., Krygier G. (2023). Quality of life in patients treated with breast cancer surgery and adjuvant systemic therapy and/or adjuvant radiotherapy in Uruguay. J. Cancer Res. Ther..

[39] Enien M.A., Ibrahim N., Makar W., Darwish D., Gaber M. (2018). Health-related quality of life. J. Cancer Res. Ther..

[40] Esgueva A.J., Noordhoek I., Kranenbarg E.M.-K., Espinosa-Bravo M., Mátrai Z., Zhygulin A., Irmejs A., Mavioso C., Meani F., González E. (2021). Health-Related Quality of Life After Nipple-Sparing Mastectomy: Results from the INSPIRE Registry. Ann. Surg. Oncol..

[41] Gillies M., Tan K., Anthony L., Miller F. (2023). Effect of Psychosocial, Behavioral, and Disease Characteristics on Health-Related Quality of Life (HRQoL) After Breast Cancer Surgery: A Cross-Sectional Study of a Regional Australian Population. Cureus.

[42] Hallberg H., Elander A., Kölby L., Hansson E. (2019). A biological or a synthetic mesh in immediate breast reconstruction? A cohort-study of long-term Health related Quality of Life (HrQoL). Eur. J. Surg. Oncol..

[43] Lagendijk M., van Egdom L., Richel C., van Leeuwen N., Verhoef C., Lingsma H., Koppert L. (2018). Patient reported outcome measures in breast cancer patients. Eur. J. Surg. Oncol..

[44] Kim M., Kim T., Moon H., Jin U., Kim K., Kim J., Lee J., Lee E., Yoo T., Noh D.-Y. (2015). Effect of cosmetic outcome on quality of life after breast cancer surgery. Eur. J. Surg. Oncol..

[45] Löfstrand J., Paganini A., Lidén M., Hansson E. (2023). Comparison of patient-reported achievements of goals and core outcomes with delayed breast reconstruction in irradiated patients: Latissimus dorsi with an implant versus DIEP. J. Plast. Surg. Hand Surg..

[46] Qin Q., Tan Q., Lian B., Mo Q., Huang Z., Wei C. (2018). Postoperative outcomes of breast reconstruction after mastectomy. Medicine.

[47] Kuroda F., Urban C., Zucca-Matthes G., de Oliveira V.M., Arana G.H., Iera M., Rietjens M., Santos G., Spagnol C., de Lima R.S. (2016). Evaluation of Aesthetic and Quality-of-Life Results after Immediate Breast Reconstruction with Definitive Form-Stable Anatomical Implants. Plast. Reconstr. Surg..

[48] Shi H., Uen Y., Yen L., Culbertson R., Juan C., Hou M. (2011). Two-year quality of life after breast cancer surgery: A comparison of three surgical procedures. Eur. J. Surg. Oncol..

[49] Sun Y., Kim S.-W., Heo C.Y., Kim D., Hwang Y., Yom C.K., Kang E. (2013). Comparison of Quality of Life Based on Surgical Technique in Patients with Breast Cancer. Ultrasound Med. Biol..

[50] Ozmen V., Ilgun S., Ozden B.C., Ozturk A., Aktepe F., Agacayak F., Elbuken F., Alco G., Ordu C., Iyigun Z.E. (2020). Comparison of breast cancer patients who underwent partial mastectomy (PM) with mini latissimus dorsi flap (MLDF) and subcutaneous mastectomy with implant (M + I) regarding quality of life (QOL), cosmetic outcome and survival rates. World J. Surg. Oncol..

[51] Spatuzzi R., Vespa A., Lorenzi P., Miccinesi G., Ricciuti M., Cifarelli W., Susi M., Fabrizio T., Ferrari M.G., Ottaviani M. (2016). Evaluation of Social Support, Quality of Life, and Body Image in Women with Breast Cancer. Breast Care.

[52] Tsai H.-Y., Kuo R.N.-C., Chung K.-P. (2017). Quality of life of breast cancer survivors following breast-conserving therapy versus mastectomy: A multicenter study in Taiwan. Ultrasound Med. Biol..

[53] Razdan S., Ahmed G.A., Vishwakarma G., Baban C., Tenovici A. (2024). Surgical and Patient-Reported Outcomes After Mastectomy and Implant-Based Prepectoral Reconstruction Using TIGR^®^ Synthetic Mesh. Cureus.

[54] Volders J.H., Negenborn V.L., Haloua M.H., Krekel N.M.A., Jóźwiak K., Meijer S., Tol P.M.v.D. (2017). Cosmetic outcome and quality of life are inextricably linked in breast-conserving therapy. J. Surg. Oncol..

[55] von Glinski M., Holler N., Kümmel S., Reinisch M., Wallner C., Wagner J.M., Dadras M., Sogorski A., Lehnhardt M., Behr B. (2022). Autologous vs. implant-based breast reconstruction after skin- and nipple-sparing mastectomy—A deeper insight considering surgical and patient-reported outcomes. Front. Surg..

[56] Janni W., Rjosk D., Dimpfl T., Haertl K., Strobl B., Hepp F., Hanke A., Bergauer F., Sommer H. (2001). Quality of Life Influenced by Primary Surgical Treatment for Stage I-III Breast Cancer?Long-Term Follow-Up of a Matched-Pair Analysis. Ann. Surg. Oncol..

[57] Han J., Grothuesmann D., Neises M., Hille U., Hillemanns P. (2009). Quality of life and satisfaction after breast cancer operation. Arch. Gynecol. Obstet..

[58] Howard M.A., Sisco M., Yao K., Winchester D.J., Barrera E., Warner J., Jaffe J., Hulick P., Kuchta K., Pusic A.L. (2016). Patient satisfaction with nipple-sparing mastectomy: A prospective study of patient reported outcomes using the BREAST-Q. J. Surg. Oncol..

[59] King M.T., Kenny P., Shiell A., Hall J., Boyages J. (2000). Quality of life three months and one year after first treatment for early stage breast cancer: Influence of treatment and patient characteristics. Qual. Life Res..

[60] Denis-Katz H.S., Ghaedi B.B., Fitzpatrick A., Zhang J. (2020). Oncological Safety, Surgical Outcome, and Patient Satisfaction of Oncoplastic Breast-Conserving Surgery With Contralateral Balancing Reduction Mammoplasty. Plast. Surg..

[61] Ticha P., Mestak O., Wu M., Bujda M., Sukop A. (2020). Patient-Reported Outcomes of Three Different Types of Breast Reconstruction with Correlation to the Clinical Data 5 Years Postoperatively. Aesthetic Plast. Surg..

[62] Szutowicz-Wydra B., Wydra J., Kruszewski W.J., Ciesielski M., Szajewski M., Walczak J., Hansdorfer-Korzon R. (2016). Same Quality of Life for Polish Breast Cancer Patients Treated with Mastectomy and Breast Reconstruction or Breast-Conserving Therapy. Pol. J. Surg..

[63] R Core Team (2024). R: A Language and Environment for Statistical Computing.

[64] Akaike H. (1974). A new look at the statistical model identification. IEEE Trans. Autom. Control.

[65] Cochran W.G. (1954). The Combination of Estimates from Different Experiments. Biometrics.

[66] Higgins J.P.T., Thompson S.G., Deeks J.J., Altman D.G. (2003). Measuring inconsistency in meta-analyses. BMJ.

[67] Higgins J.P.T., Thompson S.G., Spiegelhalter D.J. (2008). A Re-Evaluation of Random-Effects Meta-Analysis. J. R. Stat. Soc. Ser. A Stat. Soc..

[68] Egger M., Smith G.D., Schneider M., Minder C. (1997). Bias in meta-analysis detected by a simple, graphical test. BMJ.

[69] Arndt V., Merx H., Stegmaier C., Ziegler H., Brenner H. (2004). Quality of Life in Patients With Colorectal Cancer 1 Year After Diagnosis Compared With the General Population: A Population-Based Study. J. Clin. Oncol..

[70] Janz N.K., Hawley S.T., Mujahid M.S., Griggs J.J., Alderman A., Hamilton A.S., Graff J.J., Jagsi R., Katz S.J. (2011). Correlates of worry about recurrence in a multiethnic population-based sample of women with breast cancer. Cancer.

[71] Montazeri A., Vahdaninia M., Harirchi I., Ebrahimi M., Khaleghi F., Jarvandi S. (2008). Quality of life in patients with breast cancer before and after diagnosis: An eighteen months follow-up study. BMC Cancer.

[72] Corica T., Nowak A.K., Saunders C.M., Bulsara M., Taylor M., Vaidya J.S., Baum M., Joseph D.J. (2016). Cosmesis and Breast-Related Quality of Life Outcomes After Intraoperative Radiation Therapy for Early Breast Cancer: A Substudy of the TARGIT-A Trial. Int. J. Radiat. Oncol..

