# Supplementary material for: Dynamics in Quality of Life of Breast Cancer Patients Following Surgery: Systematic Review and Meta-Analysis
Source: Cancers (Basel). 2025 Sep 24;17(19):3108. doi: 10.3390/cancers17193108 (PMC12523814; doi:10.3390/cancers17193108)
Supplement: Supplementary file 1 [file cancers-17-03108-s001.zip › cancers-3866714-Supplementary/Table S1.pdf]

<table>
<tr><th>Study</th><th>n</th><th>Mean</th><th>SD</th><th>Effect</th><th>CI_lower</th><th>CI_upper</th><th>Weight</th></tr>
<tr><td>Abebe 2020 | S2 | | MastectomyAlone</td><td>86</td><td>48.25</td><td>11.79</td><td>48.25</td><td>45.76</td><td>50.74</td><td>0.70</td></tr>
<tr><td>Acil 2014 | S3 | 0-6 months | Breast_Conserving</td><td>50</td><td>68.33</td><td>18.90</td><td>68.33</td><td>63.09</td><td>73.57</td><td>0.66</td></tr>
<tr><td>Acil 2014 | S3 | 0-6 months | Mastectomy_Alone</td><td>50</td><td>46.17</td><td>27.31</td><td>46.17</td><td>38.60</td><td>53.74</td><td>0.62</td></tr>
<tr><td>Aerts 2014 | S4 | 0-6 months | Breast_Conserving</td><td>81</td><td>67.77</td><td>19.06</td><td>67.77</td><td>63.62</td><td>71.92</td><td>0.68</td></tr>
<tr><td>Aerts 2014 | S4 | 0-6 months | Breast_Conserving</td><td>73</td><td>68.66</td><td>19.94</td><td>68.66</td><td>64.09</td><td>73.23</td><td>0.68</td></tr>
<tr><td>Aerts 2014 | S4 | 7-15 months | Breast_Conserving</td><td>66</td><td>68.94</td><td>17.69</td><td>68.94</td><td>64.67</td><td>73.21</td><td>0.68</td></tr>
<tr><td>Aerts 2014 | S4 | 0-6 months | Mastectomy_Alone</td><td>68</td><td>62.85</td><td>17.32</td><td>62.85</td><td>58.73</td><td>66.97</td><td>0.68</td></tr>
<tr><td>Aerts 2014 | S4 | 0-6 months | Mastectomy_Alone</td><td>56</td><td>62.35</td><td>17.83</td><td>62.35</td><td>57.68</td><td>67.02</td><td>0.67</td></tr>
<tr><td>Aerts 2014 | S4 | 7-15 months | Mastectomy_Alone</td><td>48</td><td>66.84</td><td>15.78</td><td>66.84</td><td>62.38</td><td>71.30</td><td>0.68</td></tr>
<tr><td>Cherian 2022 | S9 | 0-6 months | Breast_Conserving</td><td>62</td><td>77.15</td><td>18.96</td><td>77.15</td><td>72.43</td><td>81.87</td><td>0.67</td></tr>
<tr><td>Cherian 2022 | S9 | 0-6 months | Breast_Conserving</td><td>62</td><td>54.57</td><td>12.77</td><td>54.57</td><td>51.39</td><td>57.75</td><td>0.70</td></tr>
<tr><td>Cherian 2022 | S9 | 7-15 months | Breast_Conserving</td><td>62</td><td>73.66</td><td>9.92</td><td>73.66</td><td>71.19</td><td>76.13</td><td>0.70</td></tr>
<tr><td>Cherian 2022 | S9 | 0-6 months | Mastectomy_Alone</td><td>76</td><td>69.63</td><td>17.78</td><td>69.63</td><td>65.63</td><td>73.63</td><td>0.68</td></tr>
<tr><td>Cherian 2022 | S9 | 0-6 months | Mastectomy_Alone</td><td>76</td><td>49.78</td><td>16.04</td><td>49.78</td><td>46.17</td><td>53.39</td><td>0.69</td></tr>
<tr><td>Cherian 2022 | S9 | 7-15 months | Mastectomy_Alone</td><td>76</td><td>69.63</td><td>11.68</td><td>69.63</td><td>67.00</td><td>72.26</td><td>0.70</td></tr>
<tr><td>Cortés-Flores 2014 | S11 | 16-30 months | Mastectomy_IBR</td><td>44</td><td>78.21</td><td>20.58</td><td>78.21</td><td>72.13</td><td>84.29</td><td>0.65</td></tr>
<tr><td>Cortés-Flores 2014 | S11 | 16-30 months | Breast_Conserving</td><td>41</td><td>85.16</td><td>17.23</td><td>85.16</td><td>79.89</td><td>90.43</td><td>0.66</td></tr>
<tr><td>Cortés-Flores 2014 | S11 | 16-30 months | Mastectomy_IBR</td><td>54</td><td>78.24</td><td>18.83</td><td>78.24</td><td>73.22</td><td>83.26</td><td>0.67</td></tr>
<tr><td>Dahlui 2023 | S12 | 0-6 months | Mastectomy_Alone</td><td>110</td><td>74.30</td><td>16.60</td><td>74.30</td><td>71.20</td><td>77.40</td><td>0.70</td></tr>
<tr><td>Dahlui 2023 | S12 | 0-6 months | Mastectomy_Alone</td><td>110</td><td>76.20</td><td>13.60</td><td>76.20</td><td>73.66</td><td>78.74</td><td>0.70</td></tr>
<tr><td>Dahlui 2023 | S12 | 7-15 months | Mastectomy_Alone</td><td>110</td><td>73.80</td><td>15.40</td><td>73.80</td><td>70.92</td><td>76.68</td><td>0.70</td></tr>
<tr><td>Dahlui 2023 | S12 | 0-6 months | Breast_Conserving</td><td>98</td><td>73.10</td><td>16.30</td><td>73.10</td><td>69.87</td><td>76.33</td><td>0.70</td></tr>
<tr><td>Dahlui 2023 | S12 | 0-6 months | Breast_Conserving</td><td>98</td><td>70.40</td><td>16.40</td><td>70.40</td><td>67.15</td><td>73.65</td><td>0.70</td></tr>
<tr><td>Dahlui 2023 | S12 | 7-15 months | Breast_Conserving</td><td>98</td><td>71.50</td><td>15.80</td><td>71.50</td><td>68.37</td><td>74.63</td><td>0.70</td></tr>
<tr><td>Harcourt 2003 | S16 | 0-6 months | Mastectomy_Alone</td><td>56</td><td>63.61</td><td>22.26</td><td>63.61</td><td>57.78</td><td>69.44</td><td>0.65</td></tr>
<tr><td>Harcourt 2003 | S16 | 0-6 months | Mastectomy_Alone</td><td>56</td><td>73.88</td><td>16.81</td><td>73.88</td><td>69.48</td><td>78.28</td><td>0.68</td></tr>
<tr><td>Harcourt 2003 | S16 | 7-15 months | Mastectomy_Alone</td><td>56</td><td>74.98</td><td>16.54</td><td>74.98</td><td>70.65</td><td>79.31</td><td>0.68</td></tr>
<tr><td>Harcourt 2003 | S16 | 0-6 months | Mastectomy_IBR</td><td>37</td><td>69.84</td><td>19.99</td><td>69.84</td><td>63.40</td><td>76.28</td><td>0.64</td></tr>
<tr><td>Harcourt 2003 | S16 | 0-6 months | Mastectomy_IBR</td><td>37</td><td>71.10</td><td>20.84</td><td>71.10</td><td>64.39</td><td>77.81</td><td>0.64</td></tr>
<tr><td>Harcourt 2003 | S16 | 7-15 months | Mastectomy_IBR</td><td>37</td><td>69.81</td><td>25.14</td><td>69.81</td><td>61.71</td><td>77.91</td><td>0.60</td></tr>
<tr><td>Hassan 2024 | S17 | | Mastectomy_Alone</td><td>154</td><td>52.30</td><td>5.20</td><td>52.30</td><td>51.48</td><td>53.12</td><td>0.71</td></tr>
<tr><td>Hassan 2024 | S17 | | Mastectomy_Alone</td><td>250</td><td>54.90</td><td>5.50</td><td>54.90</td><td>54.22</td><td>55.58</td><td>0.71</td></tr>
<tr><td>Jayasinghe 2021 | S19 | 7-15 months | Breast_Conserving</td><td>19</td><td>72.90</td><td>4.80</td><td>72.90</td><td>70.74</td><td>75.06</td><td>0.71</td></tr>
<tr><td>Jayasinghe 2021 | S19 | 7-15 months | Mastectomy_Alone</td><td>35</td><td>60.43</td><td>5.20</td><td>60.43</td><td>58.71</td><td>62.16</td><td>0.71</td></tr>
<tr><td>Konieczny 2023 | S20 | | Breast_Conserving</td><td>125</td><td>56.05</td><td>18.09</td><td>56.05</td><td>52.88</td><td>59.22</td><td>0.70</td></tr>
<tr><td>Konieczny 2023 | S20 | | Mastectomy_Alone</td><td>118</td><td>54.19</td><td>18.53</td><td>54.19</td><td>50.85</td><td>57.53</td><td>0.69</td></tr>
<tr><td>Kouwenberg 2020 | S21 | 7-15 months | Breast_Conserving</td><td>615</td><td>80.00</td><td>19.55</td><td>80.00</td><td>78.45</td><td>81.55</td><td>0.71</td></tr>
<tr><td>Kouwenberg 2020 | S21 | 7-15 months | Mastectomy_Alone</td><td>507</td><td>79.01</td><td>19.31</td><td>79.01</td><td>77.33</td><td>80.69</td><td>0.71</td></tr>
<tr><td>Kouwenberg 2020 | S21 | 7-15 months | Mastectomy_IBR</td><td>330</td><td>81.38</td><td>19.89</td><td>81.38</td><td>79.23</td><td>83.53</td><td>0.71</td></tr>
<tr><td>Kouwenberg 2020 | S21 | 7-15 months | Mastectomy_IBR</td><td>419</td><td>80.16</td><td>19.59</td><td>80.16</td><td>78.28</td><td>82.04</td><td>0.71</td></tr>
<tr><td>David Moro-Valdezate 1 2014 | S23 | 7-15 months | Mastectomy_Alone</td><td>141</td><td>66.67</td><td>16.30</td><td>66.67</td><td>63.98</td><td>69.36</td><td>0.70</td></tr>
<tr><td>David Moro-Valdezate 1 2014 | S23 | 7-15 months | Breast_Conserving</td><td>223</td><td>75.00</td><td>18.33</td><td>75.00</td><td>72.59</td><td>77.41</td><td>0.70</td></tr>
</table>

Nsaful 2024 ǀ S25 ǀ ǀ Mastectomy_Alone	162	81.25	4.69	81.25	80.53	81.97	0.71
Nsaful 2024 ǀ S25 ǀ ǀ Mastectomy_Alone	24	77.10	18.84	77.10	69.56	84.64	0.62
Nsaful 2024 ǀ S25 ǀ ǀ Breast_Conserving	67	81.25	19.86	81.25	76.49	86.01	0.67
Pačarić 2018 ǀ S27 ǀ 0-6 months ǀ Mastectomy_Alone	50	57.08	3.30	57.08	56.16	57.99	0.71
Pačarić 2018 ǀ S27 ǀ 7-15 months ǀ Mastectomy_Alone	51	61.25	14.97	61.25	57.14	65.36	0.68
Camejo 2024 ǀ S40 ǀ ǀ Mastectomy_Alone	158	83.30	24.40	83.30	79.50	87.10	0.69
Camejo 2024 ǀ S40 ǀ ǀ Mastectomy_Alone	158	66.70	24.40	66.70	62.90	70.50	0.69
Enien 2018 ǀ S47 ǀ 55-72 months ǀ Mastectomy_Alone	119	32.20	11.80	32.20	30.08	34.32	0.71
Enien 2018 ǀ S47 ǀ 55-72 months ǀ Breast_Conserving	53	27.20	11.50	27.20	24.10	30.30	0.70
Esgueva 2022 ǀ S49 ǀ 0-6 months ǀ Mastectomy_IBR	423	69.45	14.02	69.45	68.11	70.79	0.71
Esgueva 2022 ǀ S49 ǀ 7-15 months ǀ Mastectomy_IBR	423	72.97	14.02	72.97	71.63	74.31	0.71
Gillies M 2023 ǀ S56 ǀ ǀ Other	42	70.60	24.30	70.60	63.25	77.95	0.62
Hallberg H 2019 ǀ S58 ǀ 16-30 months ǀ Other	41	66.60	18.70	66.60	60.88	72.32	0.66
M. Lagendijk 2018 ǀ S62 ǀ 55-72 months ǀ Breast_Conserving	223	71.00	19.30	71.00	68.47	73.53	0.70
M. Lagendijk 2018 ǀ S62 ǀ 55-72 months ǀ Mastectomy_Alone	162	71.70	18.20	71.70	68.90	74.50	0.70
M. Lagendijk 2018 ǀ S62 ǀ 55-72 months ǀ Mastectomy_IBR	38	71.50	17.00	71.50	66.09	76.91	0.66
M. Lagendijk 2018 ǀ S62 ǀ 55-72 months ǀ Mastectomy_IBR	73	71.30	19.60	71.30	66.80	75.80	0.68
Kim 2015 ǀ S67 ǀ 7-15 months ǀ Breast_Conserving	485	63.28	15.47	63.28	61.90	64.66	0.71
Kim 2015 ǀ S67 ǀ 7-15 months ǀ Other	46	68.84	16.83	68.84	63.98	73.70	0.67
Kim 2015 ǀ S67 ǀ 7-15 months ǀ Mastectomy_Alone	86	63.50	15.52	63.50	60.22	66.78	0.69
Jonas Löfstranda 2023 ǀ S82 ǀ ǀ Mastectomy_IBR	135	61.00	26.00	61.00	56.61	65.39	0.68
Jonas Löfstranda 2023 ǀ S82 ǀ ǀ Mastectomy_IBR	118	76.00	22.00	76.00	72.03	79.97	0.69
Qin 2018 ǀ S83 ǀ 7-15 months ǀ Mastectomy_IBR	59	66.20	22.90	66.20	60.36	72.04	0.65
Qin 2018 ǀ S83 ǀ 7-15 months ǀ Mastectomy_IBR	54	65.30	16.80	65.30	60.82	69.78	0.68
Qin 2018 ǀ S83 ǀ 7-15 months ǀ Mastectomy_IBR	38	63.30	16.20	63.30	58.15	68.45	0.67
Flavia Kuroda 2016 ǀ S93 ǀ 31-54 months ǀ Mastectomy_IBR	94	84.30	13.00	84.30	81.67	86.93	0.70
Shi 2011 ǀ S95 ǀ 0-6 months ǀ Breast_Conserving	57	51.32	22.38	51.32	45.51	57.13	0.65
Shi 2011 ǀ S95 ǀ 7-15 months ǀ Breast_Conserving	57	57.61	30.38	57.61	49.72	65.50	0.61
Shi 2011 ǀ S95 ǀ 16-30 months ǀ Breast_Conserving	57	82.16	30.04	82.16	74.36	89.96	0.61
Shi 2011 ǀ S95 ǀ 0-6 months ǀ Mastectomy_Alone	83	52.19	19.80	52.19	47.93	56.45	0.68
Shi 2011 ǀ S95 ǀ 7-15 months ǀ Mastectomy_Alone	83	57.17	17.59	57.17	53.39	60.95	0.69
Shi 2011 ǀ S95 ǀ 16-30 months ǀ Mastectomy_Alone	83	76.93	27.58	76.93	71.00	82.86	0.65
Shi 2011 ǀ S95 ǀ 0-6 months ǀ Mastectomy_IBR	32	46.67	38.39	46.67	33.37	59.97	0.48
Shi 2011 ǀ S95 ǀ 7-15 months ǀ Mastectomy_IBR	32	57.07	43.80	57.07	41.89	72.25	0.43
Shi 2011 ǀ S95 ǀ 16-30 months ǀ Mastectomy_IBR	32	78.07	33.87	78.07	66.33	89.81	0.52
Young Sun1 2014 ǀ S99 ǀ 31-54 months ǀ Breast_Conserving	254	73.40	17.94	73.40	71.19	75.61	0.71
Young Sun1 2014 ǀ S99 ǀ 31-54 months ǀ Mastectomy_Alone	122	67.50	16.50	67.50	64.57	70.43	0.70
Young Sun1 2014 ǀ S99 ǀ 55-72 months ǀ Other	31	68.10	4.30	68.10	66.59	69.61	0.71
Ozmen 2020 ǀ S102 ǀ 55-72 months ǀ Mastectomy_Alone	242	66.65	17.88	66.65	64.40	68.90	0.71
Ozmen 2020 ǀ S102 ǀ 55-72 months ǀ Mastectomy_Alone	75	74.97	13.92	74.97	71.82	78.13	0.70
Spatuzzi 2016 ǀ S116 ǀ 7-15 months ǀ Breast_Conserving	72	67.60	21.10	67.60	62.73	72.47	0.67
Spatuzzi 2016 ǀ S116 ǀ 7-15 months ǀ Mastectomy_Alone	44	60.00	25.30	60.00	52.52	67.48	0.62
Spatuzzi 2016 ǀ S116 ǀ 7-15 months ǀ Mastectomy_IBR	41	69.60	19.10	69.60	63.75	75.45	0.65
Tsai 2017 ǀ S120 ǀ ǀ Breast_Conserving	217	64.90	22.00	64.90	61.97	67.83	0.70

<table>
<tr><td>Tsai 2017 | S120 | | Mastectomy_Alone</td><td>327</td><td>64.10</td><td>20.80</td><td>64.10</td><td>61.85</td><td>66.35</td><td>0.71</td></tr>
<tr><td>Razdan S 2024 | S124 | 16-30 months | Mastectomy_IBR</td><td>93</td><td>82.70</td><td>18.30</td><td>82.70</td><td>78.98</td><td>86.42</td><td>0.69</td></tr>
<tr><td>Volders 2017 | S127 | 0-6 months | Breast_Conserving</td><td>128</td><td>79.10</td><td>19.33</td><td>79.10</td><td>75.75</td><td>82.45</td><td>0.69</td></tr>
<tr><td>Volders 2017 | S127 | 0-6 months | Breast_Conserving</td><td>110</td><td>71.50</td><td>17.48</td><td>71.50</td><td>68.23</td><td>74.77</td><td>0.69</td></tr>
<tr><td>Volders 2017 | S127 | 0-6 months | Breast_Conserving</td><td>108</td><td>71.20</td><td>17.40</td><td>71.20</td><td>67.92</td><td>74.48</td><td>0.69</td></tr>
<tr><td>Volders 2017 | S127 | 7-15 months | Breast_Conserving</td><td>112</td><td>78.40</td><td>19.16</td><td>78.40</td><td>74.85</td><td>81.95</td><td>0.69</td></tr>
<tr><td>Volders 2017 | S127 | 31-54 months | Breast_Conserving</td><td>111</td><td>81.60</td><td>19.94</td><td>81.60</td><td>77.89</td><td>85.31</td><td>0.69</td></tr>
<tr><td>von Glinski 2022 | S129 | 16-30 months | Mastectomy_IBR</td><td>72</td><td>68.90</td><td>20.70</td><td>68.90</td><td>64.12</td><td>73.68</td><td>0.67</td></tr>
<tr><td>von Glinski 2022 | S129 | 31-54 months | Mastectomy_IBR</td><td>36</td><td>71.80</td><td>19.90</td><td>71.80</td><td>65.30</td><td>78.30</td><td>0.64</td></tr>
<tr><td>W. Janni 2001 | S133 | 31-54 months | Breast_Conserving</td><td>76</td><td>64.70</td><td>27.60</td><td>64.70</td><td>58.49</td><td>70.91</td><td>0.65</td></tr>
<tr><td>W. Janni 2001 | S133 | 31-54 months | Mastectomy_Alone</td><td>76</td><td>65.10</td><td>22.50</td><td>65.10</td><td>60.04</td><td>70.16</td><td>0.67</td></tr>
<tr><td>Nowicki 2015 | S137 | 0-6 months | Other</td><td>52</td><td>49.00</td><td>11.98</td><td>49.00</td><td>45.74</td><td>52.26</td><td>0.70</td></tr>
<tr><td>Nowicki 2015 | S137 | 0-6 months | Breast_Conserving</td><td>48</td><td>53.00</td><td>12.95</td><td>53.00</td><td>49.34</td><td>56.66</td><td>0.69</td></tr>
<tr><td>Songtish 2021 | S140 | | Mastectomy_Alone</td><td>30</td><td>76.11</td><td>20.96</td><td>76.11</td><td>68.61</td><td>83.61</td><td>0.62</td></tr>
<tr><td>Songtish 2021 | S140 | | Breast_Conserving</td><td>30</td><td>80.00</td><td>16.20</td><td>80.00</td><td>74.20</td><td>85.80</td><td>0.65</td></tr>
<tr><td>Lorenzo Cohen 2000 | S141 | 31-54 months | Breast_Conserving</td><td>123</td><td>74.52</td><td>17.21</td><td>74.52</td><td>71.48</td><td>77.56</td><td>0.70</td></tr>
<tr><td>Lorenzo Cohen 2000 | S141 | 31-54 months | Mastectomy_Alone</td><td>60</td><td>75.27</td><td>19.59</td><td>75.27</td><td>70.31</td><td>80.23</td><td>0.67</td></tr>
<tr><td>Domenici 2022 | S142 | 0-6 months | Mastectomy_IBR</td><td>63</td><td>61.80</td><td>14.00</td><td>61.80</td><td>58.34</td><td>65.26</td><td>0.69</td></tr>
<tr><td>Domenici 2022 | S142 | 0-6 months | Mastectomy_IBR</td><td>63</td><td>57.00</td><td>11.20</td><td>57.00</td><td>54.23</td><td>59.77</td><td>0.70</td></tr>
<tr><td>Domenici 2022 | S142 | 7-15 months | Mastectomy_IBR</td><td>63</td><td>70.30</td><td>12.20</td><td>70.30</td><td>67.29</td><td>73.31</td><td>0.70</td></tr>
<tr><td>Hejl 2021 | S144 | | Breast_Conserving</td><td>254</td><td>69.90</td><td>18.40</td><td>69.90</td><td>67.64</td><td>72.16</td><td>0.71</td></tr>
<tr><td>Hejl 2021 | S144 | | Breast_Conserving</td><td>124</td><td>61.60</td><td>17.70</td><td>61.60</td><td>58.48</td><td>64.72</td><td>0.70</td></tr>
<tr><td>Hadi 2012 | S145 | 16-30 months | Mastectomy_Alone</td><td>160</td><td>56.97</td><td>17.78</td><td>56.97</td><td>54.22</td><td>59.72</td><td>0.70</td></tr>
<tr><td>Hadi 2012 | S145 | 16-30 months | Breast_Conserving</td><td>127</td><td>66.22</td><td>12.59</td><td>66.22</td><td>64.03</td><td>68.41</td><td>0.71</td></tr>
<tr><td>Han 2010 | S146 | | Breast_Conserving</td><td>76</td><td>68.40</td><td>16.72</td><td>68.40</td><td>64.64</td><td>72.16</td><td>0.69</td></tr>
<tr><td>Han 2010 | S146 | | Mastectomy_Alone</td><td>20</td><td>64.60</td><td>15.79</td><td>64.60</td><td>57.68</td><td>71.52</td><td>0.63</td></tr>
<tr><td>Han 2010 | S146 | | Mastectomy_IBR</td><td>16</td><td>64.60</td><td>15.79</td><td>64.60</td><td>56.86</td><td>72.34</td><td>0.61</td></tr>
<tr><td>Howard 2016 | S147 | 16-30 months | Mastectomy_IBR</td><td>22</td><td>75.00</td><td>18.49</td><td>75.00</td><td>67.27</td><td>82.73</td><td>0.61</td></tr>
<tr><td>Howard 2016 | S147 | 16-30 months | Other</td><td>17</td><td>75.00</td><td>18.49</td><td>75.00</td><td>66.21</td><td>83.79</td><td>0.59</td></tr>
<tr><td>King 2000 | S151 | 0-6 months | Mastectomy_Alone</td><td>261</td><td>69.00</td><td>20.00</td><td>69.00</td><td>66.57</td><td>71.43</td><td>0.70</td></tr>
<tr><td>King 2000 | S151 | 0-6 months | Breast_Conserving</td><td>43</td><td>69.00</td><td>24.00</td><td>69.00</td><td>61.83</td><td>76.17</td><td>0.63</td></tr>
<tr><td>St Denis-Katz 2021 | S156 | 0-6 months | Other</td><td>48</td><td>78.40</td><td>19.50</td><td>78.40</td><td>72.88</td><td>83.92</td><td>0.66</td></tr>
<tr><td>St Denis-Katz 2021 | S156 | 7-15 months | Other</td><td>48</td><td>80.80</td><td>18.60</td><td>80.80</td><td>75.54</td><td>86.06</td><td>0.66</td></tr>
<tr><td>Ticha P 2020 | S157 | 55-72 months | Mastectomy_IBR</td><td>24</td><td>69.00</td><td>17.01</td><td>69.00</td><td>62.19</td><td>75.81</td><td>0.63</td></tr>
<tr><td>Ticha P 2020 | S157 | 55-72 months | Mastectomy_IBR</td><td>38</td><td>87.00</td><td>15.00</td><td>87.00</td><td>82.23</td><td>91.77</td><td>0.67</td></tr>
<tr><td>Ticha P 2020 | S157 | 55-72 months | Mastectomy_IBR</td><td>48</td><td>69.00</td><td>19.00</td><td>69.00</td><td>63.62</td><td>74.38</td><td>0.66</td></tr>
<tr><td>Szutowicz-Wydra 2016 | S160 | 7-15 months | Breast_Conserving</td><td>118</td><td>65.00</td><td>21.00</td><td>65.00</td><td>61.21</td><td>68.79</td><td>0.69</td></tr>
<tr><td>Szutowicz-Wydra 2016 | S160 | 7-15 months | Mastectomy_IBR</td><td>48</td><td>69.00</td><td>15.00</td><td>69.00</td><td>64.76</td><td>73.24</td><td>0.68</td></tr>
</table>
