# Supplementary material for: Dynamics in Quality of Life of Breast Cancer Patients Following Surgery: Systematic Review and Meta-Analysis
Source: Cancers (Basel). 2025 Sep 24;17(19):3108. doi: 10.3390/cancers17193108 (PMC12523814; doi:10.3390/cancers17193108)
Supplement: Supplementary file 1 [file cancers-17-03108-s001.zip › cancers-3866714-Supplementary/Table S2. .pdf]

Study	Methodological_Quality
Abebe 2020	Moderate methodological quality. The study had a clear objective and employed validated quality-of-life instruments appropriate for the population. Strengths include providing locally contextualized data on post-mastectomy QoL in Ethiopia. Limitations include the cross-sectional design, which precludes causal inference and longitudinal assessment of recovery trajectories, and limited generalizability beyond the Ethiopian healthcare and cultural context. Additional considerations include the relatively small sample size and reliance on self-reported outcomes, which may introduce reporting bias.
Acil 2014	Study shows acceptable methodological quality, employing standardized quality-of-life measures and a comparative design to address its research question. Limitations - modest sample size, potential confounders, and the cross-sectional approach constrain the strength and generalizability of its conclusions.
Aerts 2014	Strong methodological quality, using a prospective design. Limitations - relatively small sample size and single-country setting may limit statistical power and broader generalizability.
Cherian 2022	Prospective study design with different time-point measurements allowing assessment of changes over time. Use of validated instruments. Institutional Review Board/Ethics Committee approval and informed consent obtained. The study lacked data on socio-demographic and psychosocial variables known to impact quality of life, which limits interpretation of results.
Cortés-Flores 2014	Moderate methodological quality, offering a comparison of quality of life across three surgical procedures with a reasonably sized sample. However, its cross-sectional survey design and reliance on self-reported measures introduce bias and limit the ability to establish long-term or causal effects
Dahlui 2023	Good methodological quality, using validated quality-of-life instruments and a relatively large, diverse sample to examine surgical outcomes in Malaysian breast cancer survivors. Still, its cross-sectional design and potential cultural and healthcare system–specific influences may limit

	causal inference and generalizability to other contexts.
Harcourt 2003	Prospective, multicenter cohort with pre-operative baseline and follow-ups strengthen internal validity for temporal change. However, nonrandomized allocation to mastectomy alone vs. reconstruction (immediate or delayed) leaves room for selection bias and confounding by indication (e.g., baseline preferences, disease factors).
Hassan 2024	Fair methodological quality, providing valuable insight into quality-of-life outcomes following mastectomy with and without reconstruction in an underrepresented Iraqi population. However, its cross-sectional design, potential selection bias, and limited control of confounding factors reduce the strength and generalizability of its conclusions.
Jayasinghe 2021	Cross-sectional study evaluating post-treatment quality of life among breast cancer survivors living in Sri Lanka.<br>Women with non-metastatic breast cancer who underwent surgery between 2015–2018 and had at least one year of follow-up were invited.<br>While the sample is modest, inclusion criteria were clearly defined
Konieczny 2023	Good methodological quality, using validated instruments and a comparative approach to assess quality-of-life differences across surgical treatments. Nevertheless, its cross-sectional design and limited follow-up restrict the ability to capture long-term outcomes and establish causal relationships.
Kouwenberg 2020	Retrospective, cross-sectional patient-reported survey using multiple institutions across the Netherlands to gather long-term quality of life data up to 10 years post-surgery.
David Moro-Valdezate 2014	Methodologically sound with appropriate tools and analyses, but limited by observational design, potential selection bias, and restricted generalizability. The study provides valuable insights into predictors of QoL in breast cancer patients but should be interpreted with caution regarding causality.
Nsaful 2024	Well-reported, ethically approved, single-centre cross-sectional study using validated instruments and predefined analyses. However,

	major risks of selection and survivorship bias (large proportions not reachable or deceased; only 253/994 surgical patients ultimately analyzed) and substantial group imbalance weaken internal validity and power for between-group contrasts.
Pačarić 2018	Study uses validated QoL instruments and clear inclusion/exclusion criteria, but single-center cross-sectional design limits internal and external validity.
Camejo 2024	Moderate methodological quality, addressing an important gap by evaluating quality of life in breast cancer patients using standardized assessment tools. However, its cross-sectional design and limited sample size constrain causal interpretation and may affect the generalizability of findings beyond the local context.
Enien 2018	The study addresses an important clinical question—how surgery type and adjuvant therapy affect QoL—but lacks a prospective longitudinal design. Inclusion and exclusion criteria are reported but not very detailed.
Esgueva 2022	Multicenter registry-based study using prospective data collection. QoL assessed with validated instruments, with clear inclusion/exclusion criteria. Well-designed to evaluate outcomes after nipple-sparing mastectomy.
Gillies M 2023	Study explores associations between psychosocial, behavioral, and disease characteristics and QoL using validated instruments. However, cross-sectional design limits causal inference. Inclusion/exclusion criteria clearly reported.
Hallberg H 2019	Study compares long-term QoL outcomes between two types of mesh in immediate breast reconstruction. Cohort design allows for follow-up, though not randomized. Clear inclusion/exclusion criteria.
M. Lagendijk 2018	Strong methodological quality, utilizing validated patient-reported outcome measures and a prospective design to capture quality-of-life trajectories in breast cancer patients. Nonetheless, its findings may be limited by potential response bias and the single-country healthcare context, which could affect broader applicability.

Kim 2015	Good methodological quality, employing validated cosmetic outcome assessments and quality-of-life measures to explore their interrelationship after breast cancer surgery. Yet, its single-country setting and potential cultural influences, along with a limited ability to infer causality, somewhat restrict the generalizability of its results.
Jonas Löfstranda 2023	Study compares patient-reported outcomes in patients undergoing two types of delayed breast reconstruction. Inclusion/exclusion criteria clearly stated. Use of validated PROMs strengthens methodological quality.
Qin 2018	Moderate methodological quality, providing valuable retrospective data on postoperative outcomes after breast reconstruction using established clinical records. However, its retrospective design, potential for incomplete data, and lack of randomization limit causal inference and the generalizability of its findings.
Flavia Kuroda 2016	Study evaluates both aesthetic outcomes and patient-reported quality of life after immediate implant-based reconstruction. Clear inclusion criteria; use of validated QoL instruments supports methodological strength.
Shi 2011	Good methodological quality, using a prospective design and validated quality-of-life instruments to compare outcomes across three surgical procedures over a two-year follow-up. Nevertheless, its relatively small sample size and single-center setting may limit the generalizability of the results.
Young Sun 2014	Moderate methodological quality, employing standardized quality-of-life measures to compare outcomes across different surgical techniques. However, its cross-sectional design, limited sample size, and single-country context restrict causal inference and broader generalizability.
Ozmen 2020	Good methodological quality, using a comparative design and validated measures to assess quality of life, cosmetic outcomes, and survival rates between surgical techniques. Nonetheless, its single-center setting and potential selection bias may limit the generalizability of the findings.

Spatuzzi 2016	Moderate methodological quality, employing validated instruments to assess social support, quality of life, and body image in breast cancer patients. However, its cross-sectional design and relatively small sample size limit causal inference and the generalizability of the results.
Tsai 2017	Strong methodological quality, using a multicenter design and validated quality-of-life instruments to compare outcomes between breast-conserving therapy and mastectomy. Nonetheless, potential cultural factors and variability in treatment protocols across centers may influence generalizability beyond the Taiwanese population.
Razdan S 2024	Moderate methodological quality, providing insight into surgical and patient-reported outcomes using validated measures in implant-based prepectoral reconstruction. However, its single-center, observational design and limited follow-up reduce the strength of causal conclusions and the generalizability of findings.
Volders 2017	RCT design minimizes selection bias and allows for causal inferences. Multiple methods used to assess cosmetic outcomes enhance the robustness of findings. Longitudinal assessment of QoL provides insights into the temporal relationship between cosmetic outcomes and QoL.
von Glinski 2022	The study is a retrospective analysis of patients undergoing autologous versus implant-based breast reconstruction after skin- and nipple-sparing mastectomy. The methodology appears sound, combining surgical outcome measures with patient-reported outcomes (PROMs), which adds depth and relevance. However, being retrospective, it is subject to inherent biases such as selection bias and incomplete data capture.
W. Janni 2001	This is a comparative matched-pair study that used routinely collected follow-up QOL questionnaires to compare women who had mastectomy vs breast-conserving surgery. The matched-pair approach (matching on tumor stage) strengthens comparability for that key confounder, and the authors explicitly report balance in tumor stage, prognostic factors and adjuvant systemic therapy between groups. Because the analysis is observational (non-

	randomized, cross-sectional assessment at follow-up), the study remains prone to residual confounding (other patient factors may differ despite stage matching) and the usual limitations of non-randomized designs
Nowicki 2015	This is a cross-sectional, observational study that evaluated QOL in women following either mastectomy or breast-conserving surgery. The focus on the early postoperative period is a strength, as it captures immediate psychosocial and physical impacts. However, cross-sectional design limits causal inference, and lack of longitudinal follow-up prevents assessment of recovery trajectory.
Songtish 2021	The study investigates the correlation between body image and quality of life post-treatment. Clear objectives, but limited methodological details on sampling and control of confounders.
Lorenzo Cohen 2000	Moderate. Prospective cohort study design strengthens reliability, but limited by relatively small sample, dated instruments, and lack of standardized modern PROMs.
Domenici 2022	The study evaluates long-term outcomes of a specific reconstructive approach — muscle-sparing skin-reducing mastectomy with pre-pectoral implant placement — focusing on satisfaction and QOL. Methodological quality is moderate: prospective data collection is suggested, but the design is observational without a comparator group, limiting causal inference. Still, long-term patient-reported outcomes provide valuable insights into reconstructive practice.
Hejl 2021	Prospective non-randomized comparative study; moderate methodological quality due to prospective data collection and validated tools, but limited by lack of randomization and potential confounding.
Hadi 2012	This study evaluates QOL in women after modified radical mastectomy. The study is observational and cross-sectional, focusing on post-surgical quality of life. While it provides important data on the patient experience after MRM, the lack of a comparison group (e.g., breast-conserving surgery) limits interpretation of relative impact. The methodology is

	acceptable for descriptive purposes but limited for causal inference.
Han 2010	Moderate. Cross-sectional study with validated QoL tools, but limited by non-randomized design, potential recall bias, and absence of preoperative baseline data.
Howard 2016	Prospective observational study with validated patient-reported outcome measures. Good quality due to prospective design and standardized assessment, but lacks a control group and long-term oncological follow-up.
King 2000	This is a prospective cohort study assessing QOL at two time points (3 months and 12 months) after primary treatment for early-stage breast cancer. The study has strong methodological features: repeated measures design, relatively large sample, and evaluation of both treatment and patient-related factors influencing QOL. However, it was non-randomized, which introduces the possibility of selection bias.
St Denis-Katz 2021	Retrospective cohort study with postoperative patient-reported outcomes and clinical outcomes. Moderate methodological quality — reliable for descriptive outcomes but limited for causal inference.
Ticha P 2020	Observational, long-term follow-up study. Good methodological strength due to prospective collection of PROs, validated instruments, and 5-year follow-up. Moderate risk of bias from non-randomized group allocation
Szutowicz-Wydra 2016	This study is a cross-sectional, observational study comparing QOL in women after mastectomy with reconstruction versus breast-conserving therapy (BCT). The study's methodology is moderate: it provides relevant patient-reported outcomes using validated instruments, but cross-sectional design limits causal inference and prevents assessment of changes over time.
