# Supplementary figures and images for: Dynamics in Quality of Life of Breast Cancer Patients Following Surgery: Systematic Review and Meta-Analysis
Source: Cancers (Basel). 2025 Sep 24;17(19):3108. doi: 10.3390/cancers17193108 (PMC12523814; doi:10.3390/cancers17193108)

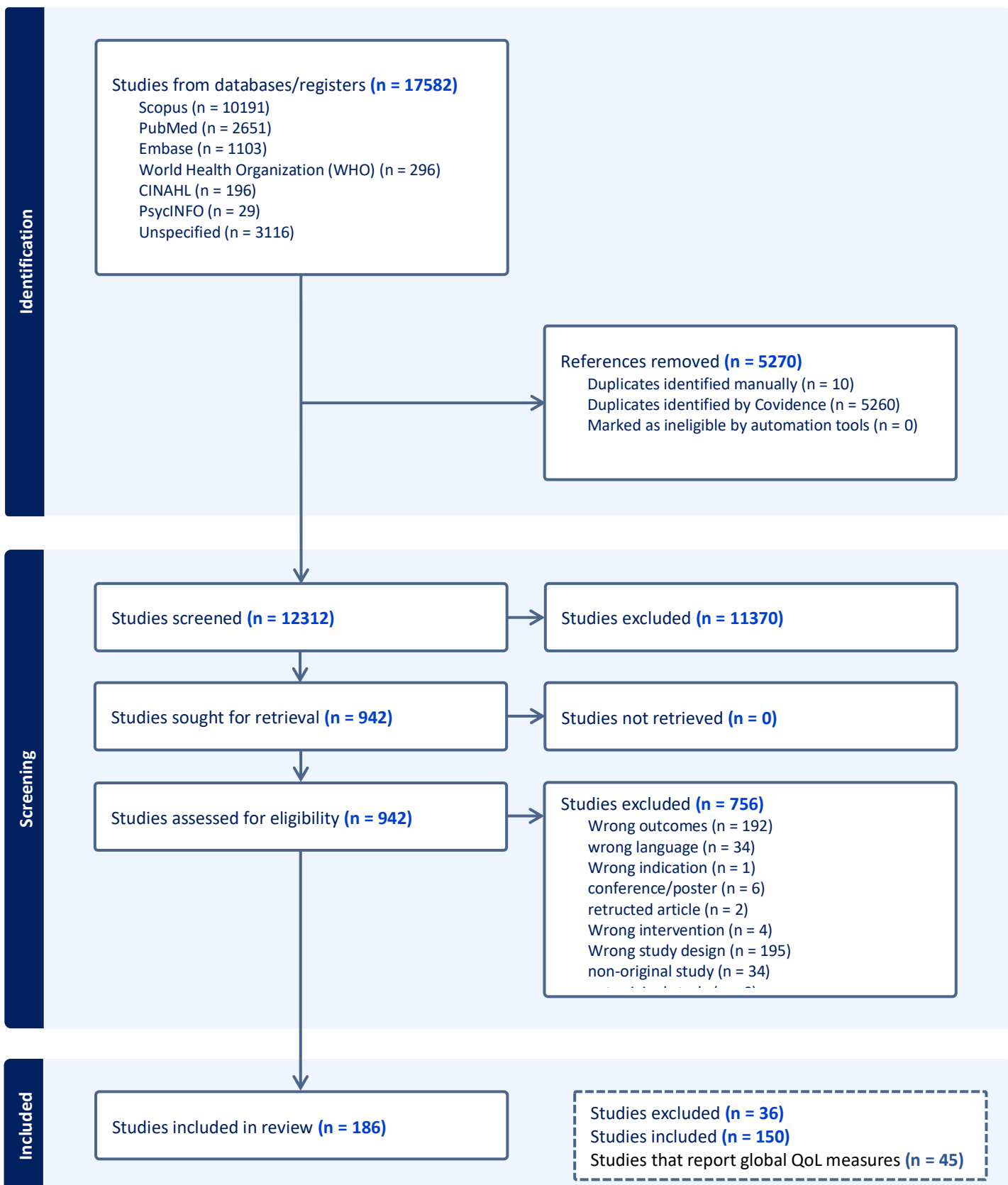

Supplement: Supplementary file 1 [file cancers-17-03108-s001.zip › cancers-3866714-Supplementary/Figure S1.pdf]
